# International Registry of *NKX2‐1*‐Related Disorders: Clinical, Genetic, and Imaging Perspectives

**DOI:** 10.1002/mds.70187

**Published:** 2026-01-19

**Authors:** Laia Nou‐Fontanet, Claudia Ravelli, Lydie Burglen, Sol Balsells Mejia, Angel Valls‐Villalba, Elies Roman Schiffels, Alice Innocenti, Beatriz Villafuerte, Ainara Salazar‐Villacorta, Vicente Quiroz, Andrea Sariego Jamardo, Giulia Bonato, Asun Díaz‐Gomez, Alexandra Afenjar, Catheline Vilain, Patricia Dumke da Silva Möller, Deyanira Garcia‐Navas Nuñez, Magdalena Krygier, Maria Judit Molnar, Łukasz Milanowski, Katrin Õunap, Micaela Pauni, Patricia Vega, Raphael Borie, Milena Villamil‐Osorio, Sanem Yilmaz, Dénes Zádori, Marta Zawadzka, Tahsin Stefan Barakat, Sebastian Neuens, Daniel de Natera‐de Benito, Dídac Casas‐Alba, Luca Soliani, Claudio M. de Gusmao, Giacomo Garone, Nicola Specchio, Miryam Carecchio, José C. Moreno, Francesca Magrinelli, Kailash P. Bhatia, Darius Ebrahimi‐Fakhari, Claudia Castiglioni, Manju Ann Kurian, João Nuno Carvalho, Roser Pons, Emmanuel Roze, Diane Doummar, Juan Darío Ortigoza‐Escobar

**Affiliations:** ^1^ Neurology Department, Coordinating Member of the ERN EpiCARE and ERN‐RND Member, Barcelona Children's Hospital Sant Joan de Déu University of Barcelona Barcelona Spain; ^2^ Pediatric Neurology Department, Neurogenetic Reference Centre for Rare Neurological Diseases Armand Trousseau Hospital APHP, Sorbonne Université Paris France; ^3^ Reference Center for Cerebellar Malformations and Congenital Diseases and Pediatric Neurogenetics Laboratory, Department of Genetics AP‐HP. Sorbonne University, Armand Trousseau Hospital Paris France; ^4^ Statistician, Sant Joan de Déu Research Foundation University of Barcelona Barcelona Spain; ^5^ Internal Medicine Department Vall d'Hebron University Hospital, Universitat Autonoma de Barcelona Barcelona Spain; ^6^ Neurology, Epilepsy and Movement Disorders Unit Bambino Gesù Children's Hospital, IRCCS, Full Member of EpiCARE Rome Italy; ^7^ Thyroid Molecular Laboratory Institute for Medical and Molecular Genetics (INGEMM), La Paz University Hospital Research Institute (IdiPAZ) Madrid Spain; ^8^ Developmental Neurosciences Department UCL GOS Institute of Child Health/Zayed Center for Research into Rare Disease in Children London UK; ^9^ Pediatric Neurology Department Boston Children's Hospital, Boston, Harvard Medical School Boston Massachusetts USA; ^10^ Pediatric Neurology Unit Marques de Valdecilla University Hospital Santander Spain; ^11^ Parkinson and Movement Disorders Unit, Department of Neuroscience, Centre for Rare Neurological Diseases (ERN‐RND) University of Padova Padova Italy; ^12^ Pediatric Neurology Unit Hospital de la Santa Creu i Sant Pau, Universitat Autònoma de Barcelona Barcelona Spain; ^13^ Department of Genetics, Hôpital Universitaire des Enfants Reine Fabiola Hôpital Universitaire de Bruxelles, Université Libre de Bruxelles Brussels Belgium; ^14^ Neurology Department Jose Alencar Brasilia Children's Hospital Brasilia Brazil; ^15^ Pediatric Neurology Unit Cáceres University Hospital Complex Cáceres Spain; ^16^ Department of Developmental Neurology Medical University of Gdansk Gdansk Poland; ^17^ Institute of Genomic Medicine and Rare Disorders Semmelweis University Budapest Hungary; ^18^ Neurology Department, Faculty of Health Sciences Medical University of Warsaw Warsaw Poland; ^19^ Institute of Clinical Medicine University of Tartu and Genetics and Personalized Medicine Clinic, Member of the ERN‐ITHACA, Tartu University Hospital Tartu Estonia; ^20^ Pediatric Neurology Department Italian Hospital of Buenos Aires Buenos Aires Argentina; ^21^ Université Paris Cité, UMR Inserm 1149, CRI, Hôpital Bichat, AP‐HP, Service de Pneumologie Allergologie et Transplantation, Centre Constitutif du Centre de Référence des Maladies Pulmonaires Rares, FHU INFIRE Paris France; ^22^ Pediatric Respiratory Department Fundación Hospital Pediátrico la Misericordia HOMI Bogotá Colombia; ^23^ Pediatric Neurology Department Ege University Medical Faculty Izmir Turkey; ^24^ Neurology Department University of Szeged Szeged Hungary; ^25^ Department of Clinical Genetics Erasmus MC University Medical Center Rotterdam Rotterdam The Netherlands; ^26^ Genetics Department, Barcelona Children's Hospital Sant Joan de Déu University of Barcelona Barcelona Spain; ^27^ IRCCS Istituto delle Scienze Neurologiche di Bologna UOC Neuropsichiatria dell'età Pediatrica Bologna Italy; ^28^ Pediatric Neurology Department University of Sao Paulo Sao Paulo Brazil; ^29^ Deparment of Neurology, Division of Movement Disorders Mass General Brigham, Harvard Medical School Boston USA; ^30^ Department of Clinical and Movement Neurosciences, UCL Queen Square Institute of Neurology University College London London UK; ^31^ Pediatric Neurology Department Clinica Meds Santiago Santiago Chile; ^32^ Faculty of Medicine Finis Terrae University Santiago Chile; ^33^ Pediatric Neurology Centro de Desenvolvimento da Criança Torrado da Silva, Garcia de Orta Hospital Almada Portugal; ^34^ Pediatric Neurology Department Children's Hospital Agia Sofia Athens Greece; ^35^ Sorbonne University, INSERM, CNRS Paris Brain Institute, AP‐HP, Institute of Neurology Paris France

**Keywords:** chorea, benign hereditary chorea, brain‐lung‐thyroid syndrome, hypothyroidism, neonatal respiratory distress syndrome, neurodevelopmental delay, *NKX2‐1*, TTF‐1

## Abstract

**Background:**

*NKX2‐1*–related disorders result from heterozygous variants in *NKX2‐1*, a gene crucial for brain, lung, and thyroid development. Although movement disorders, hypothyroidism, and neonatal respiratory distress are recognized, the full phenotype and genotype–phenotype relationships remain incompletely defined.

**Objectives:**

To delineate neurological, respiratory, and endocrine features across ages, characterize movement disorder trajectories – particularly chorea – and explore genotype–phenotype associations with clinical relevance.

**Methods:**

We conducted a multicenter, cross‐sectional study recruiting participants through referral clinicians and European networks. Standardized clinical and genetic data were captured in an electronic database and analyzed with descriptive and inferential statistics.

**Results:**

Sixty‐eight individuals (37 female; median age 16 years, range 2–60 years) were included. Motor delay was the commonest presenting feature (~60%); neonatal respiratory distress syndrome occurred in one‐third of cases. The brain–lung–thyroid triad was present in almost half. Chorea affected over 90% and began in early childhood; it was more frequent with single nucleotide variants than with deletions. Deletions are associated with better gross motor function. Frameshift or nonsense variants showed greater respiratory involvement, and variants in the exon‐3 homeobox region were associated with age‐related reduction of chorea. Neonatal respiratory distress predicted later respiratory symptoms. Greater abnormal involuntary movement severity correlated with poorer manual and gross motor function. Hypotonia and untreated hypothyroidism are associated with more severe chorea. Psychiatric comorbidity occurred in over one‐third of cases, mainly attention‐deficit/hyperactivity symptoms.

**Conclusions:**

This largest cohort to date shows early neurological onset, genotype‐specific outcomes, and frequent psychiatric comorbidity in *NKX2‐1*‐related disorders, refining clinical expectations and supporting genotype‐informed diagnosis, counseling, and management. © 2026 The Author(s). *Movement Disorders* published by Wiley Periodicals LLC on behalf of International Parkinson and Movement Disorder Society.

*NKX2‐1*‐related disorders (*NKX2‐1*‐RD), also known as brain‐lung‐thyroid syndrome or benign hereditary chorea, represent a clinically and genetically diverse group of conditions caused predominantly by pathogenic single nucleotide variants (SNVs) in the *NKX2‐1* gene, located on chromosome 14q13.^1^, ^2^ The gene encodes the thyroid transcription factor‐1 (TTF‐1), a homeodomain‐containing transcription factor essential for embryonic development and postnatal function of the brain, lungs, and thyroid gland. *NKX2‐1*‐RD typically exhibits an autosomal dominant inheritance, although *de novo* cases have also been reported.

The classic presentation of *NKX2‐1*‐RD is characterized by a triad of features: movement disorders (most commonly chorea, though dystonia, myoclonus, and ataxia may also occur), thyroid dysfunction (ranging from congenital hypothyroidism to compensated forms), and pulmonary involvement, often manifesting as neonatal respiratory distress syndrome (NRDS).^1^ The phenotypic spectrum may sometimes include one or two components of the triad or extend beyond it to additional neurological, respiratory, and endocrine manifestations. There is also emerging evidence for increased predisposition to thyroid and lung malignancies and for psychiatric comorbidities.^3^, ^4^

The NKX2‐1 protein regulates genes essential for thyroid hormone synthesis (eg, thyroglobulin, thyroperoxidase, and thyrotropin receptor),^5^, ^6^, ^7^ supports differentiation of alveolar type II cells and surfactant production in the lungs,^8^, ^9^, ^10^, ^11^ and contributes to the development of the basal ganglia, hypothalamus, and other structures involved in motor control and neuroendocrine regulation.^7^ Disruption of these functions results in multisystem disease with variable expressivity and penetrance, even among individuals with identical variants.

Despite the role of *NKX2‐1* in these disorders being established over two decades ago, important gaps remain in understanding the complete clinical spectrum, natural history, and genotype–phenotype correlations.^12^, ^13^ Current knowledge is largely derived from case reports and small cohorts, limiting insight into phenotypic diversity.^1^, ^14^, ^15^, ^16^, ^17^, ^18^, ^19^, ^20^, ^21^, ^22^, ^23^, ^24^, ^25^, ^26^, ^27^, ^28^, ^29^, ^30^ For example, while chorea is a hallmark neurological feature, its longitudinal course is poorly defined^31^; the longest reported follow‐up spans 24.5 years and includes 28 individuals.^29^ The frequency and severity of respiratory and thyroid manifestations also vary, and correlations with genotype are only beginning to emerge.^32^, ^33^ Recent studies have expanded the genetic spectrum to include deletions, mobile element insertions, changes in conserved non‐coding regions, and variants in regulatory genes (eg, *PAX9*, *MBIP*) that may influence phenotype.^34^, ^35^

This heterogeneity complicates diagnosis and management. Symptom overlap with other neurodevelopmental, movement, and endocrine disorders often delays diagnosis. In addition, the unpredictable evolution of symptoms makes prognosis and long‐term treatment planning challenging.

To address these gaps, this study analyzed the largest multicenter cohort of genetically confirmed *NKX2‐1*‐RD to date. Through detailed clinical, genetic, and neuroimaging assessments, we aimed to define the full range of neurological, respiratory, and endocrine manifestations across ages, characterize the progression of movement disorders – particularly chorea – and identify genotype–phenotype associations that can guide diagnosis, prognostication, and targeted management strategies.

## Patients and Methods

### Study Design and Population

This multicenter, cross‐sectional, observational study included individuals with *NKX2‐1*‐RD and a confirmed genetic diagnosis. Participants were recruited through referral physicians and international outreach via several platforms, including the European Reference Network for Rare Neurological Diseases (ERN‐RND), the European Reference Network for Rare Malformation Syndromes, Intellectual and Other Neurodevelopmental Disorders (ERN‐ITHACA), the Spanish Society of Pediatric Neurology (SENEP), the Pediatric Movement Disorders Special Interest Group (SIG) of the International Parkinson and Movement Disorder Society (MDS), and the Facebook *NKX2‐1* patient group. Additional recruitment was achieved through workshops, scientific congresses, and direct referrals from colleagues.

All study data were collected and managed in a REDCap (Research Electronic Data Capture) database for the International *NKX2‐1* Registry,^36^, ^37^ hosted at Hospital Sant Joan de Déu, Barcelona, Spain, between October 2023 and October 2024. In total, 40 specialists from 29 centers across 17 countries contributed to the registry. Clinical data were obtained from medical records and de‐identified to ensure confidentiality.

Clinical and ancillary data were collected using a standardized registry protocol and analyzed following predefined criteria. Core variables included demographic information, perinatal history, neurological findings, systemic features, and genetic results. Movement‐disorder phenomenology was assessed by subspecialists at each site, and severity was graded using the Abnormal Involuntary Movement Scale (AIMS) when available. Brain magnetic resonance imaging (MRI) and endocrine assessments were reviewed locally. Genetic testing followed site‐specific protocols and American College of Medical Genetics and Genomics (ACMG)/Association for Molecular Pathology (AMP) classification guidelines (*NKX2‐1*, transcript NM_001079668.3). Statistical analyses included descriptive, univariate, and multivariate models using appropriate nonparametric tests (Spearman, Mann–Whitney, Kruskal–Wallis, Chi‐squared/Fisher) with *P* < 0.05 considered significant.

A detailed description of data collection procedures, operational definitions (eg, gait abnormalities, cognitive impairment), and statistical methods is provided in Supplementary Material, Methods in Data S1.

## Results

### Demographic and Neurological Features

**FIG. 1:**
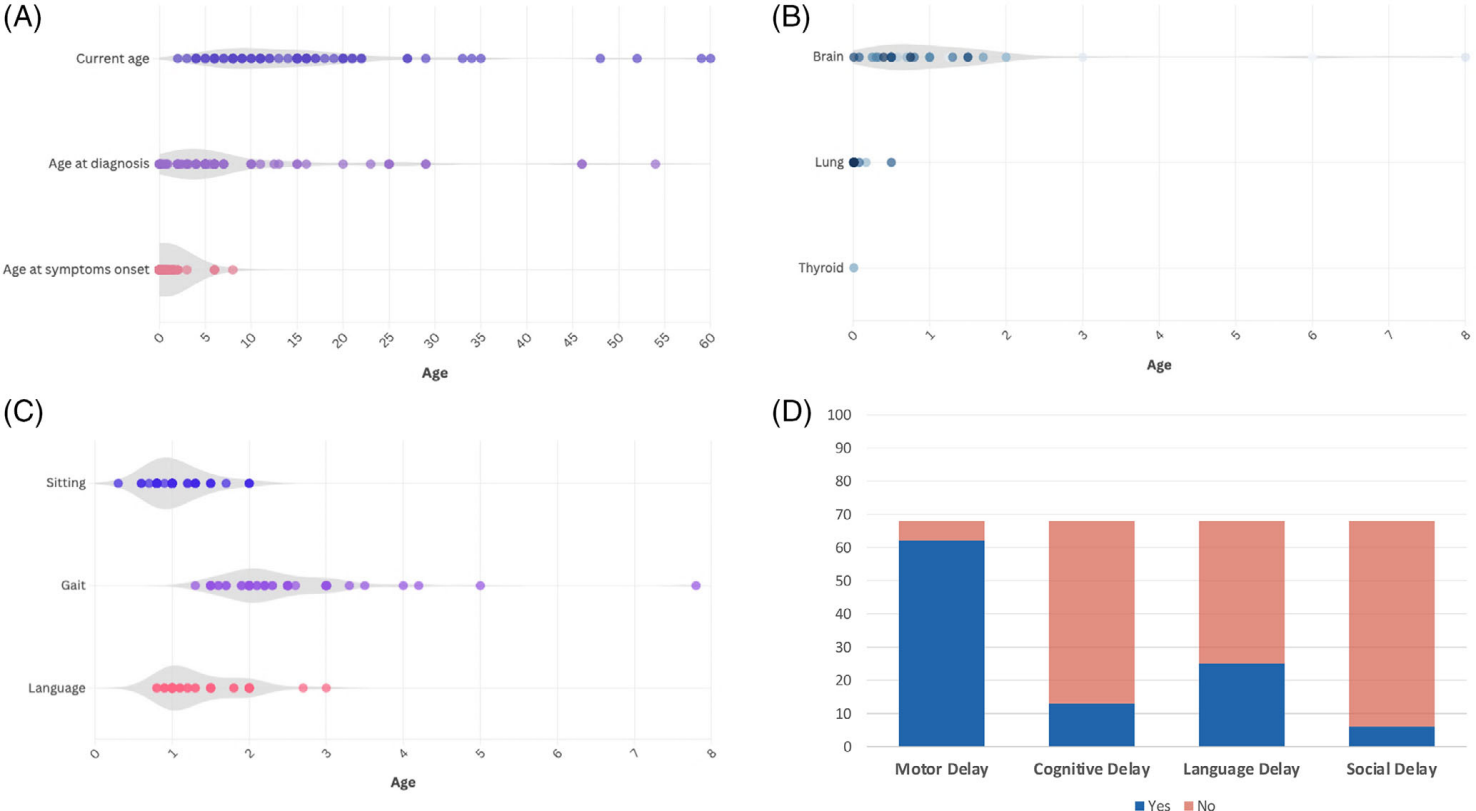
Clinical characterization of individuals with *NKX2‐1*‐related disorders (*NKX2‐1*‐RD). (A) Violin plot depicting current age (in years), age at *NKX2‐1*‐RD diagnosis, and age at onset of first symptoms. (B) Violin plot showing the age (in years) at onset of brain, lung, and/or thyroid symptoms. (C) Violin plot illustrating the age (in years) at attainment of sitting, gait, and language milestones. (D) Column chart showing the percentage of individuals with *NKX2‐1*‐RD presenting motor, cognitive, language, and social developmental delays. [Color figure can be viewed at wileyonlinelibrary.com]

Sixty‐eight individuals with *NKX2‐1*‐RD were included; 37 (54.4%) were female. Median age at last follow‐up was 16 years (range 2–60 years) (Fig. 1A). Most were referred by neurologists (86.8%). Eighty‐five percent were followed in Europe (notably France and Spain) and 15% were in the Americas. One neonatal death due to NRDS occurred. Fourteen cases had been previously published.^5^, ^22^, ^28^, ^29^, ^38^, ^39^, ^40^, ^41^, ^42^, ^43^

**FIG. 2:**
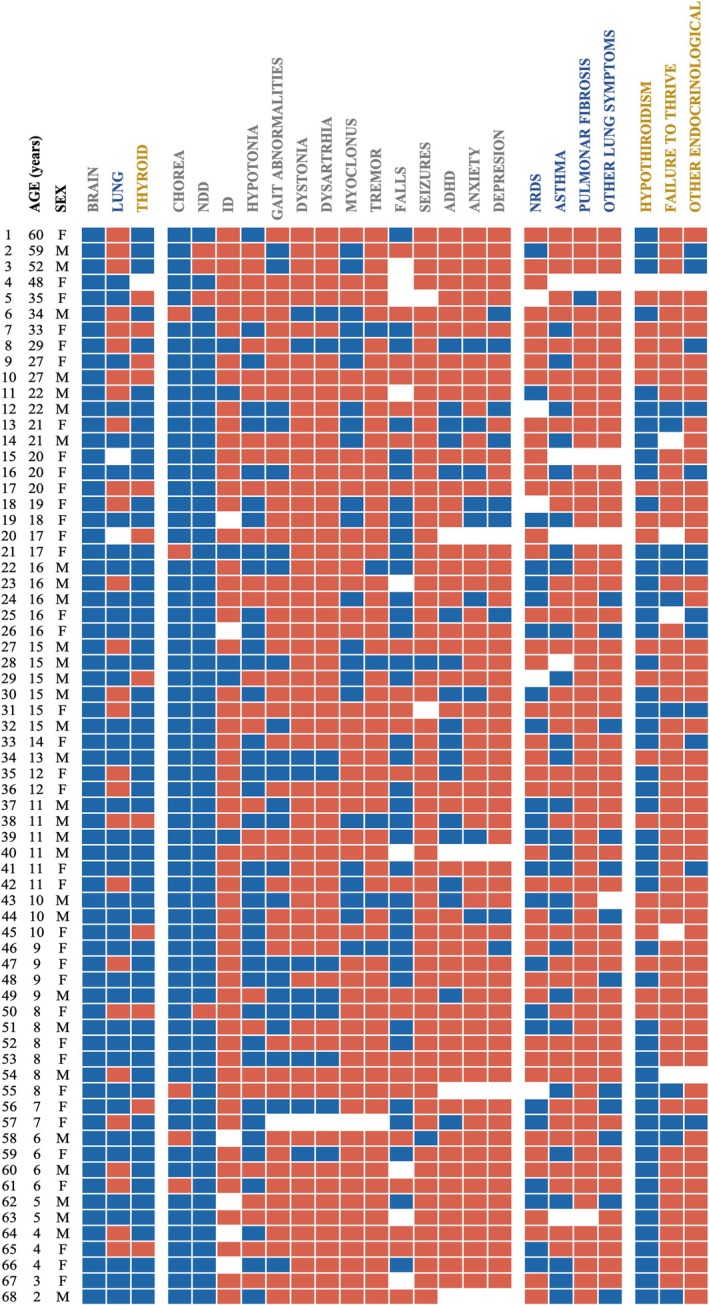
Phenotypic heatmap of individuals with *NKX2‐1*‐related disorders (*NKX2‐1‐*RD). The heatmap illustrates the presence or absence of the classical brain–lung–thyroid triad, together with various neurological, respiratory, and endocrinological symptoms, categorized by age and sex. Red indicates absence, blue indicates presence, and white represents missing data. NDD, neurodevelopmental delay; ID, intellectual disability; NRDS, neonatal respiratory distress syndrome; ADHD, attention‐deficit hyperactivity disorder; F, female; M, male. [Color figure can be viewed at wileyonlinelibrary.com]

Median age at first symptom was within the first year (median 0.87 years, interquartile range [IQR] 0–8 years) (Fig. 1A). Neurological symptoms predominated at onset: motor delay was most frequent (41.8%), followed by global developmental delay and hypotonia; chorea or gait problems were initial in a minority. Respiratory onset occurred in 34.3% (mostly NRDS) and endocrine onset was rare. Only half (50.8%) fulfilled the full brain–lung–thyroid triad; 40% had dual‐system involvement (most often brain–thyroid), and 9.2% showed isolated brain involvement (Fig. 1B). A phenotypic heatmap for age and sex is shown in Figure 2.

Perinatal complications were common. NRDS was reported in 35.5% (median onset 4.5 hours after birth). About half of those with NRDS required invasive ventilation; others received noninvasive support or oxygen. Additional neonatal issues (25.81%) included hyperbilirubinemia, non‐reassuring fetal status (eg, abnormal fetal heart rate patterns), and late prematurity. Among term infants, median birthweight was 3135 g; newborn screening was abnormal in 22.7%, all subsequently diagnosed with hypothyroidism (Supplementary Table S1).

**FIG. 3:**
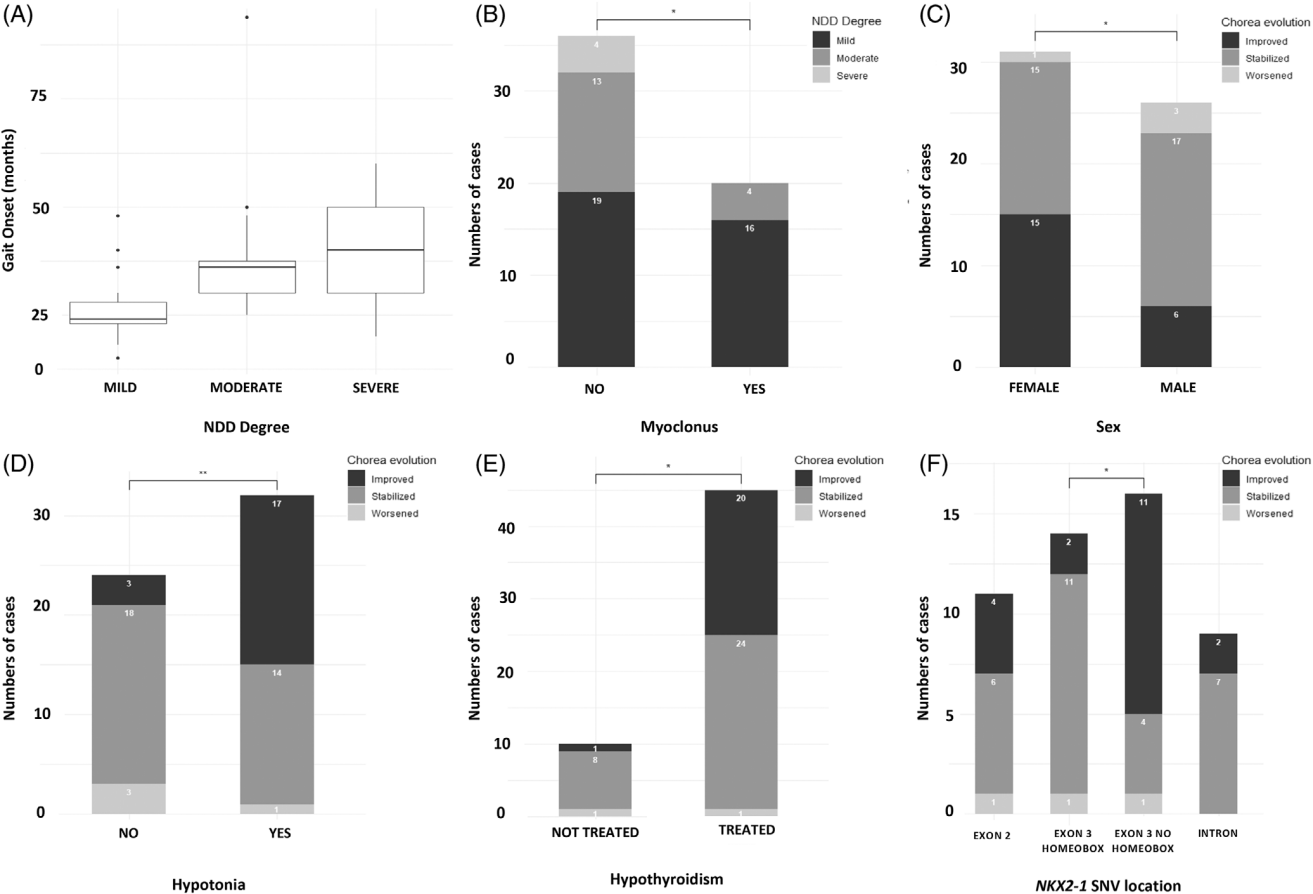
Clinical associations in individuals with *NKX2‐1*‐related disorders (*NKX2‐1‐*RD), focusing on neurodevelopmental delay (NDD) and chorea. (A) Age at gait onset by NDD severity. Boxplot illustrating gait onset age (in months) in individuals with mild, moderate, and severe NDD. Median, interquartile range, and outliers are shown, indicating a significant correlation between delayed gait onset and NDD severity. (B) Distribution of NDD severity in individuals with and without myoclonus. Bar chart showing the number of cases with mild, moderate, or severe NDD, stratified by presence (Yes) or absence (No) of myoclonus. A significant difference in NDD severity was observed between groups. (C) Chorea evolution by sex. Boxplot showing age at chorea onset (months) by sex, with a trend toward earlier onset in females that was not significant in multivariable models. (D) Chorea evolution by presence of hypotonia. Stacked bar chart comparing outcomes of chorea (improved, stabilized, worsened) in individuals with and without hypotonia. A significant difference was observed. (E) Chorea evolution by presence of treated hypothyroidism. Stacked bar chart showing chorea evolution in individuals with and without treated hypothyroidism. A significant difference was observed. (F) Chorea evolution by location of *NKX2‐1* single nucleotide variants (SNV). Stacked bar chart comparing chorea evolution across individuals with variants in different gene regions. A significant difference was observed. Statistical significance: *P < 0.05, **P < 0.01, ***P < 0.001.

Neurodevelopmental delay (NDD) was present in 94% (64/68): mild 62.1%, moderate 31.0%, and severe 6.9% (when graded). Motor delay was nearly universal (91.2%), whereas speech (36.8%), other cognitive (19.1%), and social (8.8%) impairments were less frequent (Fig. 1C,D). Independent sitting occurred at 12.4 months on average; autonomous gait at 29.5 months. Only 10.7% walked before 19 months, and later gait onset correlated with greater NDD severity (Spearman *r* = 0.524, *P* < 0.001) (Fig. 3A).

Chorea affected 92.7% (Supplementary Table S2). It began early (median 2.00 years; 93% before age 6 years). Chorea was predominantly generalized (80.6%). Among patients with non‐generalized chorea, the most frequently affected body regions were the arms (66.7%), head (22.2%), and legs (11.1%). Chorea was more frequent with *NKX2‐1* SNVs than with deletions (96.6% vs. 75%, Fisher's exact test *P* = 0.067). Females showed earlier onset when univariate Mann–Whitney U test analysis was performed, but this information was not retained after adjustment in the multivariable linear regression.

Evolution of chorea could be classified in those with ≥2 documented assessments; given the heterogeneous and non‐uniform observation intervals across patients, results are reported as categorical evolution (improved/stable/worsened) rather than as time‐to‐change. Over time, chorea stabilized in 57.1%, improved in 35.7%, and worsened in 7.1%. Females appeared to have better outcomes in unadjusted analysis (Fisher's exact test *P* = 0.035) (Fig. 3C), but this did not persist in multivariable logistic regression controlled by possible confounders. Two clinical factors associated with a worse chorea course were hypotonia (Fisher's exact test *P* = 0.002) and untreated hypothyroidism (Fisher's exact test *P* = 0.032) (Fig. 3D,E). By genotype, homeobox exon‐3 SNV tended to remain stable, whereas non‐homeobox SNV more often improved (Fisher's exact test *P* = 0.039) (Fig. 3F). On the AIMS rating scale (n = 52), 25% had minimal, 57.7% mild, and 17.3% moderate chorea.

Other motor features were frequent (82.4%): hypotonia 57.4%, dystonia 46.3%, gait abnormalities 34.3%, myoclonus 32.8%, dysarthria 14.9%, and tremor 9.0%. Frequent falls were reported in 61% of those queried. Myoclonus was associated with dystonia (51.6% vs. 17.1%, χ^2^ = 8.790, df = 2, *P* = 0.007) and with gait abnormalities/dysarthria (70% vs. 28.6%, χ^2^ = 7.299, df = 2, *P* = 0.026). Myoclonus was uncommon before the second decade but present in ~49% thereafter (χ^2^ = 6.271, df = 2, *P* < 0.05) (Fig. 3B). Intellectual disability was documented in 11.5%; most others were in the borderline–mild range.

Functional scales showed mild impairment overall (Gross Motor Function Classification System [GMFCS] I–II in 95%; Manual Ability Classification System [MACS] I–II in 88%). Deletions were uniformly associated with better gross motor function (GMFCS I) than SNV (Fisher's exact test *P* = 0.045). Males had worse manual ability (higher MACS scores) than females (Fisher's exact test *P* = 0.047).

### Systemic Features

Respiratory symptoms occurred in 57.6%; recurrent wheezing and bronchospasm consistent with reactive airway disease were most common (42.9%). NRDS predicted later respiratory disease (87% vs. 45%, χ^2^ = 13.816, df = 2, *P* = 0.001). The average age at the onset of recurrent wheezing and bronchospasm consistent with reactive airway disease was ~1.2 years. Recurrent infections were reported in seven individuals; interstitial lung disease in two; and one adult had unclassifiable pulmonary fibrosis. Only about one‐fifth underwent spirometry or chest computed tomography; diffusion capacity was rarely assessed. Treatments included inhaled steroids, bronchodilators, and azithromycin. Frameshift/nonsense SNVs were associated with higher respiratory involvement than other SNVs (χ^2^ = 8.270, df = 2, *P* = 0.016).

Hypothyroidism was highly prevalent (82%; median diagnosis age was 1.54 years, nearly half in infancy). Median thyroid‐stimulating hormone (TSH) at diagnosis was elevated, and median free thyroxine (T4) was low‐normal. Thyroid ultrasound (available in half the cases) showed congenital hypoplasia in about one‐third. Most hypothyroid individuals (85%) received levothyroxine, with heterogeneous dosing; precise data distinguishing congenital versus compensated cases were incomplete.

Beyond the triad, 19.7% had other endocrine issues: growth hormone deficiency (9.1%), hypogonadotropic hypogonadism (4.6%), and isolated cases of hypopituitarism or early puberty. Anthropometrics were generally within reference ranges, and thyroid dysfunction did not associate with failure to thrive or short stature.

Psychiatric comorbidity was common (over one‐third). Attention‐deficit/hyperactivity symptoms were most frequent, followed by anxiety and depression. Myoclonus was significantly associated with anxiety and depression (χ^2^ = 7.967, df = 2 *P* < 0.01 and χ^2^ = 12.776, df = 2, *P* < 0.01, respectively). No cases of autism spectrum disorder or obsessive–compulsive disorder were recorded. Treatments included methylphenidate (often continued at the last follow‐up), guanfacine, or atomoxetine in isolated cases, and selective serotonin reuptake inhibitors or antipsychotics when indicated.

About 31% reported additional non‐triad features (eg, fatigue, urinary incontinence, joint hyperlaxity, mild humoral immunodeficiency); oncological processes were not observed. Review of 13 facial photographs did not reveal a consistent dysmorphic pattern.

### Genetic Diagnosis and Neuroimaging

Median age at genetic diagnosis was 5 years. Testing modalities included exome sequencing (clinical or whole; 79%), microarray‐based comparative genomic hybridization (8%), whole‐genome sequencing (3%), and others. Most individuals (83.8%) harbored *NKX2‐1* SNV; the remainder had deletions or regulatory‐region disruptions (eg, *MBIP*, *PAX9*). Among SNV, nonsense (40.4%) and frameshift (28.1%) were most frequent, followed by splicing (15.8%) and missense (17.8%). SNVs are distributed across exon 2, exon 3, and splice regions; all missense variants are clustered within the homeobox domain. Most nonsense variants also lay within the homeobox; only one frameshift was located there (distal). Two related individuals had an Alu retrotransposition in exon 3. Eight individuals (11.8%) had deletions (two megadeletions and six microdeletions); five encompassed *NKX2‐1* with other genes, and three spared *NKX2‐1* but included regulatory partners (*MBIP* in two and *PAX9* in one). One individual had a translocation involving the *NKX2‐1* region (Supplementary Fig. S1, Table S3).

According to ACMG, 57.8% of SNVs/copy number variants (CNVs) were pathogenic, 40.6% likely pathogenic, and 1.6% variant of unknown significance (VOUS). One case (I7) harbored a heterozygous 14q13.3 microdeletion (arr[GRCh37] 14q13.3(36,722,498–36,790,795)x1; 68.3 kb) encompassing *MBIP*. The clinical presentation was concordant with *NKX2‐1*–RD. According to the ClinGen CNV rubric (evidence codes 1A, 3A, 4 N), this CNV remains classified as a VOUS trending toward likely pathogenic at the time of resubmission. Approximately half of the variants were *de novo*; among inherited variants with known parents, maternal transmission predominated. Seven multiplex families are represented, though parental data were incomplete in most, limiting analyses of penetrance and intrafamilial variability.

Brain MRI (performed in 80.6%) was normal in 67.9%. Reported abnormalities included corpus callosum dysgenesis, delayed myelination, and cystic lesions (7.6%), such as Rathke cleft cysts and an arachnoid cyst. Other findings were empty sella, Chiari I malformation, and non‐specific white matter changes.

### Treatment and Management

**FIG. 4:**
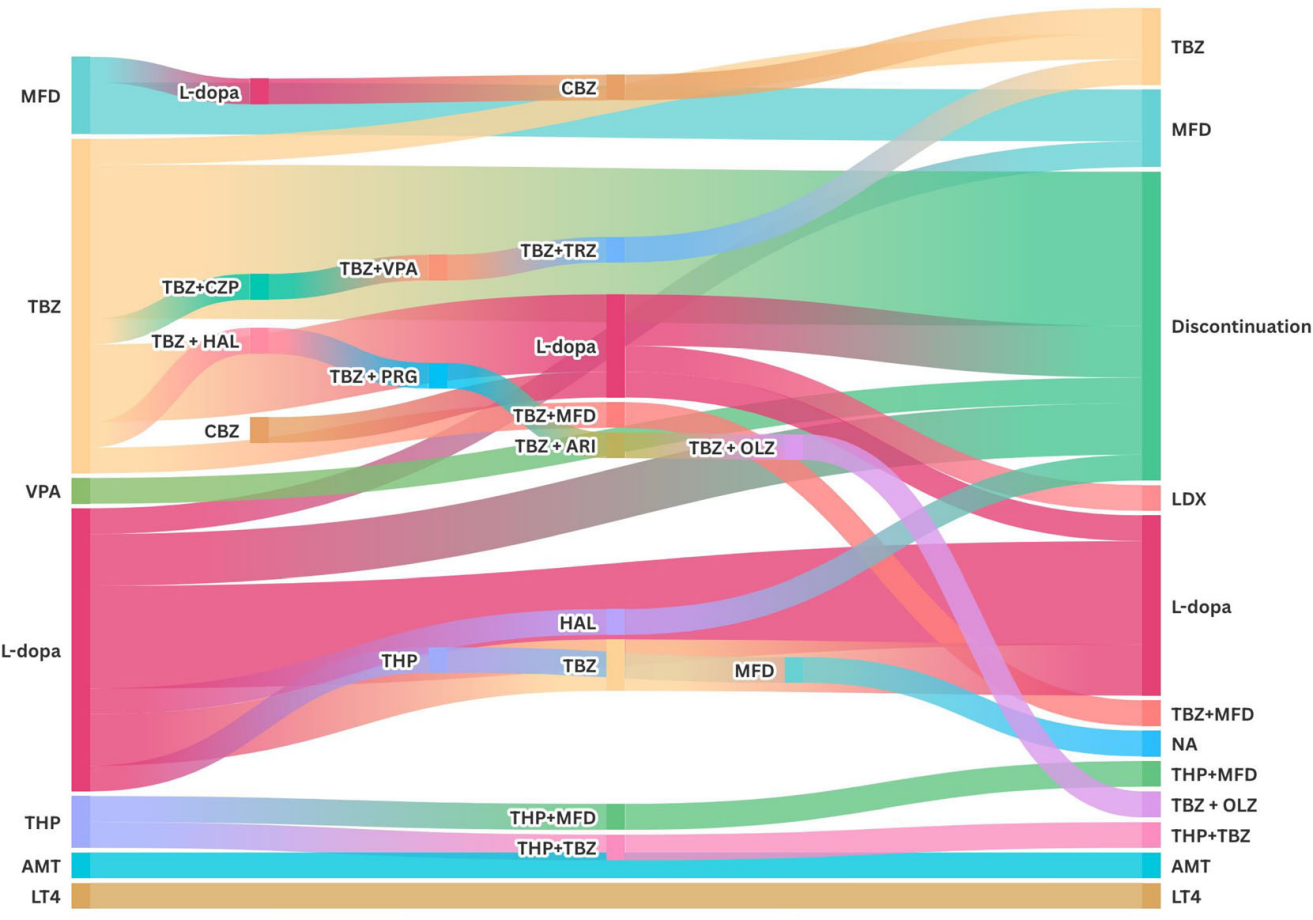
Sankey diagram showing the different treatments received for chorea in individuals with *NKX2‐1*‐related disorders (*NKX2‐1‐*RD). MFD, methylphenidate; CBZ, carbamazepine; TBZ, tetrabenazine; CZP, clonazepam; VPA, valproate; TRZ, trazodone; HAL, haloperidol; PRG, pregabalin; ARI, aripiprazole; OLZ, olanzapine; LDX, lisdexanfetamine; THP, trihexyphenidyl; NA, Not available; AMT, amantadine; LT4, levothyroxine. [Color figure can be viewed at wileyonlinelibrary.com]

Movement disorder‐directed therapies were used in 35/63 (55.6%). The most frequently prescribed agents were tetrabenazine (54.3% of treated patients), levodopa (45.7%), and methylphenidate (22.9%). Tetrabenazine was initiated at a median age of 6.5 years and was often discontinued due to limited benefit or adverse effects. Levodopa and methylphenidate provided moderate benefit in a subset of patients. At the last follow‐up, approximately half of the treated individuals remained on monotherapy. One patient with dystonia underwent globus pallidus internus (GPi) deep brain stimulation with a favorable response (I7). Details are provided in Supplementary Table S2 and Figure 4.

Care was multidisciplinary (neurology, pulmonology, and endocrinology), with periodic assessment of motor function, respiratory status, and thyroid hormones, alongside neurodevelopmental and psychiatric monitoring (Supplementary Table S4).

A more detailed description of the results can be found in the Supplementary Material in Data S1.

## Discussion

Our findings broaden the clinical and genetic understanding of *NKX2‐1*–RD and challenge reliance on the classical brain–lung–thyroid triad for diagnosis. Novel contributions include the delineation of early motor delay as the most typical presenting sign, genotype–phenotype associations that inform motor and respiratory outcomes, and the identification of NRDS as a predictor of later respiratory morbidity. Together, these insights refine prognosis and support a multidisciplinary care model.

Motor delay within the first year was frequent and often preceded other manifestations, occurring independently of global neurodevelopmental delay.^1^, ^29^ Although most individuals had some neurodevelopmental difficulty, cognitive and social deficits were less prominent than motor impairment. The combination of early motor delay with hypothyroidism, early pulmonary disease, or hypotonia should heighten suspicion for *NKX2‐1*‐RD.

Chorea affected the vast majority of patients and usually began in early childhood, then stabilized or improved in most cases – distinct from the progressive course typical of metabolic or neurodegenerative chorea. Complete resolution was not observed here, although Grass et al. reported it in a subset.^29^ The observed stabilization or improvement from childhood onward likely reflects natural history in a non‐degenerative disorder, although treatment effects (eg, tetrabenazine, levodopa, or thyroid replacement) may modulate the clinical course in individual cases; our registry was not designed to isolate causal treatment effects.

Importantly, improvement in chorea did not parallel other triad features: hypothyroidism persisted and required treatment, and respiratory disease could remain stable or emerge later (eg, interstitial lung disease or asthma). The mechanism of chorea improvement remains unclear; age‐related increases in tone within a non‐degenerative motor system is a plausible explanation. Although myoclonus was more frequent after the second decade, we did not observe a statistical association with chorea improvement.

Although all the individuals in this cohort had neurological symptoms, this finding should not be interpreted as universal to *NKX2‐1‐*RD. Referral pathways mainly involved pediatric neurologists, which likely biased ascertainment toward individuals with neurological presentations and may have missed those with isolated respiratory or thyroid involvement.

NRDS occurred in about one‐third of cases and strongly predicted later respiratory symptoms, extending prior observations.^44^ A systematic review of 148 individuals showed a respiratory spectrum from NRDS to asthma and interstitial lung disease; nonsense *NKX2‐1* SNVs have even been linked to lung cancer.^45^ These features are consistent with *NKX2‐1* haploinsufficiency affecting surfactant biology, while the endocrine profile underscores its role in thyroid development.^7^ Treatments ranged from oxygen and ventilation to transplantation, and long‐term outcomes were heterogeneous.^45^

Hypothyroidism was highly prevalent and often diagnosed in infancy, reinforcing the need for early screening. Individuals with mild or compensated hypothyroidism who were not started on levothyroxine showed more severe chorea compared with those receiving treatment or without hypothyroidism, based on retrospective clinical evaluations. This association should be interpreted with caution, because thyroid status was not systematically evaluated before and after treatment initiation, and treatment decisions depended on local clinical judgement. Although thyroid dysfunction in *NKX2‐1*‐RD is well recognised,^32^, ^33^ our cohort revealed underuse of levothyroxine, despite established benefits of early therapy. This, together with inconsistent neonatal TSH screening and diagnostic delays, argues for standardized endocrine protocols. We recommend routine thyroid testing in children with neurodevelopmental disorders and lifelong monitoring in all individuals with *NKX2‐1*‐RD. Treatment of compensated hypothyroidism should be considered carefully given its association with chorea severity, acknowledging the need for confirmation in prospective studies.

Additional motor features – hypotonia, dystonia, and myoclonus – and their interactions (including links between myoclonus, dystonia, and milder neurodevelopmental delay) extend the phenotype beyond chorea. We did not observe consistent dysmorphic features, including in deletion cases, and no malignancies occurred in this relatively young cohort.

Only about half fulfilled the full triad; 40% had dual‐system involvement – most often brain–thyroid – and ~9% presented with isolated brain disease. These data reinforce variable expressivity in line with prior reports.^1^, ^29^, ^30^, ^46^ The predominance of neurological presentations likely reflects recruitment through neurology and may underrepresent isolated pulmonary or thyroid disease.

Individuals with SNVs were more likely to develop chorea than those with deletions, while deletions – regardless of size or inclusion of neighboring genes – were associated with better gross motor function. Variants in the exon‐3 homeobox domain were associated with more stable chorea, whereas non‐homeobox variants tended to improve. Frameshift and nonsense variants correlated with increased respiratory involvement, with a near‐significant trend for splicing variants. These associations build on earlier work and provide practical guidance for prognosis and follow‐up.^43^

The stabilization or improvement of chorea distinguishes *NKX2‐1*‐RD from progressive disorders such as DYT‐*HPCA*, CHOR/DYT‐*ADCY5*, and *GNAO1*‐related disease.^47^, ^48^, ^49^, ^50^ Although there have been descriptions of chorea evolving into myoclonus,^29^ our cross‐sectional design prevents us from drawing definitive conclusions about longitudinal trajectories. Correlations between functional motor scales (higher AIMS with worse MACS and GMFCS) and delayed gait with neurodevelopmental severity are novel and suggest that early motor milestones serve as prognostic markers.

We identified Alu retrotransposition in two related individuals and deletions affecting regulatory genes rather than *NKX2‐1* itself, expanding the spectrum beyond prior reports and underscoring the value of comprehensive genomic testing.^5^

## Clinical Implications

**FIG. 5:**
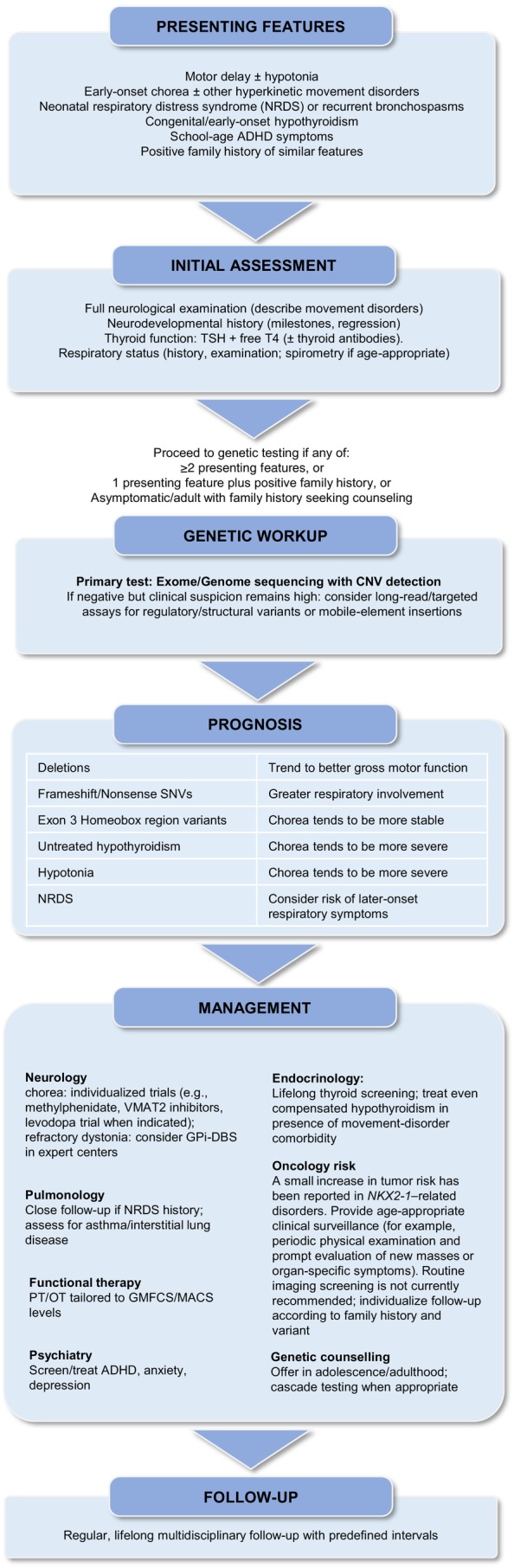
Practical diagnostic and management workflow for *NKX2‐1*–related disorders. Algorithm summarizing the clinical approach from initial presentation to genetic work‐up, prognosis, and follow‐up. Key recommendations include early recognition of motor delay or chorea with neonatal respiratory distress or hypothyroidism, early exome/genome sequencing, and regular multidisciplinary follow‐up (neurology, pulmonology, endocrinology, psychiatry, rehabilitation, and genetics). NRDS, neonatal respiratory distress syndrome; ADHD, attention‐deficit/hyperactivity disorder; TSH, thyroid‐stimulating hormone; T4, free thyroxine; CNV, copy number variant; SNV, single nucleotide variant; VMAT2, vesicular monoamine transporter 2; GPi, globus pallidus internus; DBS, deep brain stimulation; PT, physiotherapy; OT, occupational therapy; GMFCS, Gross Motor Function Classification System; MACS, Manual Ability Classification System. [Color figure can be viewed at wileyonlinelibrary.com]

*NKX2‐1*‐RD should be considered in neonates with disproportionate NRDS and in infants with isolated motor delay, particularly when hypotonia or early chorea are present, even without overt thyroid disease (Fig. 5). Early genetic testing should interrogate SNVs, deletions, and, when the exome/genome is negative but the phenotype is compelling, regulatory regions and mobile‐element insertions. Prognosis should incorporate genotype and sex (eg, better gross motor outcomes with deletions; worse manual ability in males). Differential diagnosis includes *NHLRC2*, which can mimic the respiratory and thyroid phenotype but typically presents with dystonia rather than chorea.^51^ The limited and variable response of chorea to medication argues for individualized therapeutic trials, ideally stratified by genotype and sex.

The genotype–phenotype patterns suggest that functional domains of *NKX2‐1* differentially influence neurodevelopment, motor control, and respiratory regulation. The relatively milder motor phenotype in deletions versus SNVs may reflect partial preservation of regulatory networks, a hypothesis for future molecular work. Associations between myoclonus, dystonia, and psychiatric comorbidities hint at broader circuit dysfunction, potentially across basal ganglia‐thalamo‐cortical loops, consistent with contemporary models linking movement disorders and psychiatry.^52^

The cross‐sectional design limits causal inference and likely biased recruitment toward neurologically presenting cases; improvement or stabilization was inferred retrospectively from clinical documentation rather than quantified through standardized longitudinal scales, restricting precision in assessing temporal evolution. Although this is the largest cohort to date, subgroup analyses remain underpowered. Management varied across centers, potentially affecting outcomes. The median age at genetic diagnosis suggests a diagnostic delay that may obscure early trajectories. Under‐representation of pulmonology expertise and interstitial lung disease cases limits generalizability to those subgroups. Severity and longitudinal changes in chorea and myoclonus were derived from retrospective expert clinical documentation and AIMS ratings when available. Because AIMS lacks validation in *NKX2‐1*–RD and standardized serial scales were inconsistently applied at fixed intervals, measurement variability is possible, preventing time‐to‐event analyses. Additionally, because the registry relied on clinician notes rather than prospective standardized assessments, the longitudinal evolution of chorea may be subject to documentation bias.

Prospective, longitudinal cohorts with standardized neurological, pulmonary, and endocrine assessments are needed to validate predictors (eg, NRDS for later pulmonary disease) and to define natural history. Mechanistic studies contrasting homeobox versus non‐homeobox SNVs and assessing regulatory disruptions may reveal therapeutic targets. Genotype‐ and sex‐stratified treatment studies, together with routine psychiatric screening, should inform evidence‐based care.

In summary, *NKX2‐1*‐RD is clinically heterogeneous, but chorea, NRDS, and hypothyroidism remain defining features. Motor delay is a frequent early sign that may presage chorea. By mapping genotype‐specific outcomes and early clinical predictors, this study offers a framework for genotype‐informed diagnosis, counseling and management, reinforcing the need for coordinated care across neurology, pulmonology, endocrinology, and psychiatry.

## Author Roles

(1) Research Project: A. Conceptualization, B. Methodology, C. Data Curation, D. Investigation, E. Data Analysis, F. Visualization; (2) Statistical Analysis: A. Design, B. Execution, C. Review and Critique; (3) Manuscript Preparation: A. Writing of the First Draft, B. Review and Editing; (4) Other: A. Supervision.

L.N.F.: 1B, 1C, 1D, 1E, 1F, 3A, 3B.

S.B.M., A.V.‐V., E.R.S.: 1C, 1D, 1E, 1F, 3B.

C.R., B.V., A.S.‐V., C.V., R.B., S.N., D.N.d.B., D.C.‐A., L.S., C.M.d.G., G.G., F.M., C.C., R.P., E.R., D.D.: 1C, 1D, 3B.

L.B., A.I., V.Q., A.S.J., G.B., A.D.‐G., A.A., P.D.d.S.M., D.G.‐N.N., M.K., M.J.M., L.M., K.O., M.P., P.V., M.V.‐O., N.S., M.C., J.C.M., K.B., D.E.‐F., M.K., J.N.C.: 1C, 1D, 3B.

J.D.O.‐E.: 1A, 1B, 1C, 1D, 1F, 3B, 4A.

## Financial Disclosures and Conflicts of Interest

Author disclosures are available in the Supporting Information.

## Financial Disclosures for the Previous 12 Months

The authors declare that there are no additional disclosures to report.

## Supporting information


**Data S1.** Methods.

**Data S2.** Supporting Information.

**Table S1.** Perinatal characteristics and anthropometric data in individuals with *NKX2‐1*‐related disorders.

**Table S2.** Clinical course and treatment response of chorea in individuals with *NKX2‐1‐*related disorders.

**Table S3.** Genetic and in silico data of *NKX2‐1* variants in the study cohort.

**Table S4.** Frequency and percentage of *NKX2‐1*‐related disorders individuals followed up by each medical specialist.

**Figure S1.** Distribution of 45 *NKX2‐1*‐single nucleotide variants relative to the functional domains of *NKX2‐1* isoform 2 (RefSeq NM_003317.3). Distal and proximal promoters are indicated by arrows. Functional domains are shown in dark grey: TN (tinman domain), HD (homeodomain), and NK2 (NK2‐specific domain). Nonsense variants (orange), frameshift variants (blue); missense variants (green); splicing – non‐coding variants (yellow), and Alu retrotransposition events (grey).

**Video S1.** Clinical phenotype in adults with *NKX2‐1‐*related disorder. This video presents three adult individuals with *NKX2‐1‐*related disorder, shown in order of increasing age to illustrate the phenotypic spectrum across adulthood. Individual 1: Mild chorea involving the upper limbs and neck, more evident during arm extension and sustained posture. Individual 2: Mild chorea, predominantly affecting the trunk and lower limbs. Individual 3: Mild generalized chorea, with increased amplitude during voluntary movements. (https://figshare.com/s/4a7dac1757e993d72836).

**Video S2.** Clinical phenotype in adolescents with *NKX2‐1‐*related disorder. This video presents five adolescent individuals with *NKX2‐1‐*related disorder, shown in order of increasing age to illustrate the phenotypic spectrum across adulthood. Individual 13: Mild chorea of the upper limbs and shoulders, and of the lower limbs when extending the arms forward and attempting to maintain both arms raised. Individual 22: Orofacial chorea when protruding the tongue. Chorea of the neck, head, and trunk when attempting to remain still. Bilateral upper limb chorea with arms extended forward, during the supination–pronation manoeuvre, and when holding both arms in front of the chest. Clumsiness during hand tapping. Mild gait disturbance. Slow and effortful handwriting with choreic movements. Individual 29: Choreic movements of the upper and lower limbs when attempting to remain still. Distal upper limb athetosis also observed. Individual 34: Chorea of the upper and lower limbs at rest, and when extending the arms forward or attempting to keep the fingers still. Dorsiflexion of the metacarpophalangeal joints. Impaired coordination on the finger‐to‐nose test. Gait interference, with difficulty walking on heels and toes. Unable to maintain posture during the Romberg test, though no falls occur. Impaired tracing on the Archimedes spiral. Slow and effortful handwriting. Individual 35: Choreic movements of the upper limbs. “Touchdown sign” present when raising the arms. Gait difficulties also noted. (https://figshare.com/s/5e61d1610f534fa2266f).

**Video S3.** Clinical phenotype in children with *NKX2‐1*‐related disorders. This video presents four child individuals with *NKX2‐1‐*related disorder, shown in order of increasing age to illustrate the phenotypic spectrum across adulthood. Individual 37: Choreic movements of the upper limbs, shoulders, and lower limbs at rest. Mild to moderate upper limb chorea when extending the arms forward, exacerbated by the supination–pronation maneuver. Handwriting is slow. Gait is impaired, with torsion dystonia of the foot. Individual 40: Upper limb chorea when attempting to maintain the arms extended forward. Gait is slightly better than in the previous patient, with the ability to perform one‐legged hopping. Handwriting is slightly more agile. Individual 62: Chorea of the upper and lower limbs, with dystonic posturing of both feet. Requires assistance to walk. Individual 67: Chorea of the upper and lower limbs, visible particularly in the feet when extending the arms forward. Chorea is moderate. Difficulty with rapid hand opening and closing. Impaired performance on the finger‐to‐nose test. Requires support for walking, with knee hyperflexion. (https://figshare.com/s/f43cb6659a1ed71c49f4).

**Video S4.** Longitudinal follow‐up of Individual 57 with *NKX2‐1*‐related disorder. Individual 57: Longitudinal follow‐up. In the initial part of the video, upper limb and trunk chorea is observed. Gait is impaired, with difficulty performing motor tasks and clumsiness; falls may occur, although not captured in the video. Later, chorea persists, with marked difficulty and slowness when squatting or performing fine motor tasks, such as cracking a nut. Further along, difficulty running is observed, with falls. Gait improves with the use of an anterior walker, although hyperflexion of the joints is noted. Chorea improves slightly with the use of orthoses, but dystonic posturing of both feet is evident. Chorea improves over time. (https://figshare.com/s/31c513dc830f61eb124e).

## Data Availability

The data that support the findings of this study are available on request from the corresponding author. The data are not publicly available due to privacy or ethical restrictions.
