# Supplementary material for: International Registry of NKX2‐1‐Related Disorders: Clinical, Genetic, and Imaging Perspectives
Source: Mov Disord. 2026 Jan 19;41(4):889–900. doi: 10.1002/mds.70187 (PMC13067339; doi:10.1002/mds.70187)
Supplement: Supplementary file 1 — Data S1. Methods. [file MDS-41-889-s001.docx]

**Clinical, laboratory, and ancillary data**

Demographic and clinical information was collected using a standardized protocol. Data included sex, country of origin, and age at last follow-up. Participants were categorized into eight age groups based on age at evaluation: neonatal period (first 28 days of life), infancy (28 days–1.9 years), early childhood (2–5 years), late childhood (6–11 years), adolescence (12–17 years), young adulthood (18–39 years), adulthood (40–60 years), and older adulthood (>60 years).

Perinatal history included pregnancy and delivery complications, as well as birth anthropometrics. Neurological assessment covered the presence and type of neurological features (e.g., hypotonia, developmental delay, gait abnormalities) and movement disorder phenomenology, including chorea, dystonia, myoclonus, and tremor. Chorea severity was assessed by movement-disorders specialists at each clinical visit. When available, severity was quantified using the Abnormal Involuntary Movement Scale (AIMS); for patients without AIMS, severity was categorized (minimal, mild, moderate) based on standardized descriptors in clinical documentation. Although AIMS was originally designed for drug-induced movement disorders and has not been specifically validated for *NKX2-1*-RD, it provided a standardized reference when available.

Myoclonus and tremor were diagnosed clinically by movement-disorder specialists. Electrophysiological studies (e.g., EMG) were not systematically performed, although they can support the characterization of jerky movements in selected cases. For each individual, the longitudinal trajectory of chorea was classified as improved, stable, or worsened by comparing serial clinical notes and functional status across visits (e.g., interference with fine/gross motor tasks, falls, school/day-to-day functioning). Classification required documentation from at least two time points; otherwise evolution was recorded as not assessable. Because follow-up intervals varied across sites and patients, no uniform time window was imposed.

Response to movement-disorder medications was assessed based on treating clinician documentation, reflecting perceived changes in chorea severity and functional impact (e.g., motor control, falls, and daily activities), as standardized longitudinal response scales were not uniformly applied in this retrospective cohort.

Gait abnormalities were defined as impairments in gait stability, trajectory or coordination documented by a movement disorders specialist, including wide-based gait, frequent falls, dystonic posturing during ambulation or delayed acquisition of independent walking beyond 18 months. Cognitive impairment was assessed clinically, supported by evidence of educational support needs or enrolment in special education programs. Brain MRI findings were reviewed locally by the treating neurologist and/or neuroradiologist at each participating site; centralized image re-evaluation was not performed. Endocrinological evaluation included thyroid hormone levels and treatment history. In cases of mild or compensated hypothyroidism, clinicians sometimes deferred levothyroxine therapy; these patients were classified as “not treated” for subgroup comparisons. Respiratory evaluation included pulmonary function testing, history of respiratory infections, and oxygen dependency. Additional systemic manifestations were also documented. Gross motor function and manual ability were graded using the Gross Motor Function Classification System (GMFCS) and the Manual Ability Classification System (MACS).

Genetic analysis was performed at each participant’s institution, following site-specific protocols and ethical guidelines. Methods included karyotyping, targeted gene panels, whole-exome sequencing (WES), whole-genome sequencing (WGS), optical genome mapping (OGM), and chromosomal microarrays. Variant classification followed the American College of Medical Genetics and Genomics/Association for Molecular Pathology (ACMG/AMP) guidelines, using the *NKX2-1* transcript NM_001079668.3 for nomenclature. Deletions were grouped by size into microdeletions (<5 Mb), macrodeletions ( 5-10 Mb), and megadeletions (>10 Mb). Patients with deletions outside *NKX2-1* were included if their phenotype overlapped with brain–lung–thyroid syndrome and the genomic variant was predicted to affect *NKX2-1* expression.

When available, facial photographs were reviewed by a clinical geneticist to identify dysmorphic features and provide additional phenotypic data.

**Statistical Analysis**

A descriptive analysis was performed. Descriptive statistics were calculated for numerical variables, which are described with median and range due to lack of normality. QQ plots were used to assess normality for these variables. Frequency tables were generated for categorical variables, which are described with frequencies and percentages. The valid percentages were reported (presence of a predetermined variable out of the total number of non-missing data).

Univariate analysis was conducted to explore relationships between variables. For pairs of numerical variables, Spearman’s correlation was applied. For comparisons of two groups, Mann–Whitney U-test was used for numerical or ordinal variables; for more than two groups, Kruskal–Wallis test was applied, with pairwise Mann–Whitney U-tests and Bonferroni–Holm adjustment as post-hoc analysis when results were significant. Associations between categorical variables were assessed using Chi-squared test or Fisher’s exact test when any expected cell count was fewer than five.

Multivariate analysis was performed when univariate analysis revealed statistically significant associations. Depending on the nature of the dependent variable, multiple linear regression, binary logistic regression, or ordinal logistic regression was applied.

All tests were two-tailed, and statistical significance was set at p < 0.05. Analyses were performed using R (v. 4.3.2) in RStudio (v. 2022.02).

**Data availability**

The data that support the findings of this study are available from the corresponding authors, upon reasonable request.
