# Supplementary material for: International Registry of NKX2‐1‐Related Disorders: Clinical, Genetic, and Imaging Perspectives
Source: Mov Disord. 2026 Jan 19;41(4):889–900. doi: 10.1002/mds.70187 (PMC13067339; doi:10.1002/mds.70187)
Supplement: Supplementary file 3 — Table S1. Perinatal characteristics and anthropometric data in individuals with NKX2‐1‐related disorders. [file MDS-41-889-s006.docx]

**Supplementary Table 1. Perinatal characteristics and anthropometric data in individuals with *NKX2-1-*related disorders**

| **ID** | **NRDS (hours at presentation) , maximum respiratory support required** | **Other remarkable perinatal history, symptomatology** | **Weight (grams)** | **Length (cm)** | **Head Circumference  (cm)** | **Newborn Screening Test** |
| --- | --- | --- | --- | --- | --- | --- |
| 1 | - | - | NA | NA | NA | Normal |
| 2 | - | - | 3500 | 55 | NA | Not Performed |
| 3 | - | - | NA | NA | NA | Not Performed |
| 4 | + (NA), NA | NA | NA | NA | NA | NA |
| 5 | - | - | NA | NA | NA | Not Performed |
| 6 | - | - | NA | NA | NA | Not Performed |
| 7 | NA | NA | NA | NA | NA | NA |
| 8 | - | - | NA | NA | NA | Not Performed |
| 9 | - | - | 2960 | 49 | 33.5 | Not Performed |
| 10 | - | - | NA | NA | NA | Not Performed |
| 11 | - | - | NA | NA | NA | Not Performed |
| 12 | - | - | 3570 | 48 | 37 | NA |
| 13 | + (0), Conventional IMV | +, NINHB requiring phototherapy | 3400 | 48 | 35 | Abnormal |
| 14 | NA | NA | NA | NA | NA | NA |
| 15 | - | - | 3340 | NA | NA | Normal |
| 16 | - | - | NA | NA | NA | Normal |
| 17 | - | - | NA | NA | NA | NA |
| 18 | - | - | 3265 | 48.5 | 35 | Not Performed |
| 19 | - | NA | 2635 | 46 | 32 | Not Performed |
| 20 | NA | NA | NA | NA | NA | NA |
| 21 | + (4), High-Frequency IMV | +, hypotonia | 2800 | 49 | 34 | Normal |
| 22 | - | +, LFWB (APGAR 3/6/9) | 4410 | 54 | 37 | Normal |
| 23 | - | - | NA | NA | NA | Normal |
| 24 | + (NA), COT | +, Late Prematurity (34 weeks) | NA | NA | NA | Not Performed |
| 25 | + (0), High-Frequency IMV | +, NINHB requiring Phototherapy and Pulmonary Artery Hypertension | 3880 | 48.5 | 37.5 | NA |
| 26 | + (NA), Non-IMV | - | NA | NA | NA | Normal |
| 27 | - | - | 3000 | 48 | NA | Abnormal |
| 28 | + (0), High-Frequency IMV | - | 3095 | 49.5 | 35 | NA |
| 29 | - | - | 3110 | NA | NA | Normal |
| 30 | - | - | 3910 | 52 | 38 | Normal |
| 31 | NA | - | 2860 | 50 | 34 | Not Performed |
| 32 | + (NA), Non-IMV | - | 2350 | NA | NA | NA |
| 33 | - | - | 2190 | 46.5 | 32 | Abnormal |
| 34 | + (7), HFOT | - | 3410 | 54 | 37.5 | Normal |
| 35 | - | - | 3300 | 50 | 34 | Abnormal |
| 36 | - | - | 2520 | 46.5 | 33 | Normal |
| 37 | - | - | 3680 | 52 | 35 | Normal |
| 38 | - | - | 3215 | 50 | 35 | Normal |
| 39 | + (1), Non-IMV | - | NA | NA | NA | Abnormal |
| 40 | + (6), Conventional IMV | - | 3160 | 50 | 35.5 | Abnormal |
| 41 | + (0), COT | +, LFWB and Emergency Caesarean (Membranes Rupture) | 4170 | NA | 34.5 | Normal |
| 42 | - | - | 2480 | NA | 31.5 | Normal |
| 43 | + (0), Conventional IMV | +, LFWB | 3500 | 51 | 36 | NA |
| 44 | - | +, Streptococcus septicaemia (1 day of life) | 3620 | 53 | 36 | Not Performed |
| 45 | + (0), Non-IMV | - | 3350 | 51.3 | 34 | Normal |
| 46 | - | - | NA | NA | NA | Normal |
| 47 | - | +, IGR | 2090 | 44.5 | 31 | Not Performed |
| 48 | - | - | 2650 | NA | NA | Normal |
| 49 | + (48), NA | - | 2920 | 49.5 | 34 | Normal |
| 50 | - | - | 3200 | 45 | 34 | Normal |
| 51 | - | - | 3720 | 50 | 35 | Normal |
| 52 | + (2), COT | +, NINHB requiring phototherapy | 2740 | 49 | 32 | Normal |
| 53 | + (NA), NA | +, NINHB requiring phototherapy | 2800 | NA | NA | Abnormal |
| 54 | - | - | 3000 | 54 | NA | Normal |
| 55 | - | + | NA | NA | NA | NA |
| 56 | - | - | 2760 | 47.7 | 33.3 | Normal |
| 57 | NA | NA | NA | NA | NA | NA |
| 58 | + (0), High-Frequency IMV | - | 2420 | NA | NA | Normal |
| 59 | + (1), Non-IMV | - | 2870 | 50 | 32.5 | Normal |
| 60 | - | - | 3380 | 48 | 35 | Normal |
| 61 | - | +, Central Apnoea | 3250 | 49 | 35 | Normal |
| 62 | - | +, Eating Difficulties | 3420 | 50.5 | 34 | Abnormal |
| 63 | + (5), High-Frequency IMV | - | 2270 | 41 | 33.5 | Normal |
| 64 | + (2), Conventional IMV | - | NA | NA | NA | Normal |
| 65 | - | - | 2850 | 55 | 32 | Normal |
| 66 | - | - | 2750 | 46.5 | 33.5 | Normal |
| 67 | + (NA), Non-IMV | +, NINHB requiring phototherapy and Pneumonia | NA | NA | NA | Abnormal |
| 68 | + (0), High-Frequency IMV | +, Pulmonary Artery Hypertension and Late Prematurity (35.4 weeks) | NA | NA | NA | Abnormal |

Individual Identification (ID), Neonatal Distress Respiratory Syndrome (NDRS), Yes (+), No (-), Non available (NA), Invasive Mechanical Ventilation (IMV), Extracorporeal membrane oxygenation (ECMO), High Flow Oxygen Therapy (HFOT),Conventional Oxygen Therapy (COT), Non-isoimmune neonatal hyperbilirubinemia requiring  phototherapy (NINHB), Loss of Fetal Well-Being (LFWB), Intrauterine Growth Restriction (IGR).
