# Supplementary material for: International Registry of NKX2‐1‐Related Disorders: Clinical, Genetic, and Imaging Perspectives
Source: Mov Disord. 2026 Jan 19;41(4):889–900. doi: 10.1002/mds.70187 (PMC13067339; doi:10.1002/mds.70187)
Supplement: Supplementary file 4 — Table S2. Clinical course and treatment response of chorea in individuals with NKX2‐1‐related disorders. [file MDS-41-889-s011.docx]

**Supplementary Table 2. Clinical course and treatment response of chorea in individuals with *NKX2-1*-related disorders**

| **ID** | **Age at Onset (years)** | **Chorea Evolution** | **Age at Change (years)** | **AIMS** | **Chorea Pharmacological Treatment (dosage); Age at starting treatment (years), Duration of the treatment (years). Effect, cessation/continuation.** | | | | |  |
| --- | --- | --- | --- | --- | --- | --- | --- | --- | --- | --- |
| 1 | NA | = | NA | 2 |  |  |  |  |  |  |
| 2 | 6 | = | 6 | 2 | MFD (NA); NA, NA. Without effect, stopped due to LOI | THP (NA); NA, NA. NA, continued | TBZ (25mg/12h); NA, NA. NA, continued |  |  |  |
| 3 | 6 | -- | 12 | 4 | TBZ (25mg/8h); 44, 2. Much improvement, stopped due to non-specified causes | | | | |  |
| 4 | NA | = | NA | NA |  |  |  |  |  |  |
| 5 | 2 | = | 10 | 2 |  |  |  |  |  |  |
| 7 | 1 | NA | NA | NA |  |  |  |  |  |  |
| 8 | 3 | = | 12 | 3 | TBZ (50mg/day); 27, 1.5. Improvement, continued | Haloperidol (0.5mg/day); 26, 0.5. Without effect, stopped due to AE and LOI | Pregabalin (NA);26,0.5.Without effect, stopped due to LOI | Aripiprazole (10mg/day); 27, 0.5. Worsening, stopped due to AE and LOI | Olanzapine (25mg/day); 27, 0.5. A little improvement, continued |  |
| 9 | 0.70 | ++ | 9 | 3 |  |  |  |  |  |  |
| 10 | 1.50 | = | 10 | 2 | Amantadine (100mg/day); 18, 9. A little improvement, continued | | | | |  |
| 11 | 10 | ++ | 14 | 3 | VPA (NA); 7,7. Much improvement, stopped due to non-specified causes | | | | |  |
| 12 | 2 | ++ | 20 | 2 |  |  |  |  |  |  |
| 13 | NA | = | NA | 2 | TBZ (NA); 20, 0. NA, stopped due to AE | | | | |  |
| 14 | NA | = | 15 | 2 |  |  |  |  |  |  |
| 15 | 3.50 | ++ | NA | 2 | MFD (NA); NA, NA. Without effect, stopped due to LOI | L-dopa (1mg/kg/day); NA, 0. NA, continued | TBZ (25mg/day); NA, NA. Improvement, continued | CBZ (NA); NA, NA. Without effect, stopped due to LOI |  |  |
| 16 | 2.67 | ++ | NA | 2 | L-dopa (3 mg/kg/day); 3.5, 1. Improvement, stopped due to non-specific causes | | MFD (57 mg/day); NA, NA. Improvement, continued | |  |  |
| 17 | 2 | = | NA | 3 | TBZ (NA); 16, 0.3. Without effect, stopped due to AE | | | | |  |
| 18 | 1 | = | 12 | 2 |  |  |  |  |  |  |
| 19 | 0.92 | = | 11 | 2 | TBZ (5mg/12h); 5, 0.1. Without effect, stopped due to AE | L-dopa (3mg/kg/day); 5, 3. Much improvement, stopped due to adhesion problems. Restarted at 17 years old without improvement, 3 mg/kg/day. Suspended at 2 months and a half due to AE | | | |  |
| 20 | NA | NA | NA | NA | L-dopa (NA); NA, NA. NA, stopped due to non-specified causes | | | | |  |
| 21 | NA | NA | NA |  |  |  |  |  |  |  |
| 22 | 1.25 | ++ | 5 | 3 |  |  |  |  |  |  |
| 23 | 6 | = | 7 | 3 | TBZ (25mg/day); 13, 0.2. Without effect, stopped due to LOI and adhesion problems | | | | |  |
| 24 | 6 | -- | 10 | 2 | TBZ (6.25mg/day); 11, 1. Without effect, stopped due to LOI and adhesion problems | | | | |  |
| 25 | 4 | ++ | 12 | 2 | TBZ (5mg/12h); 5,7. Improvement, stopped due to non-specific causes | | | | |  |
| 26 | 2.50 | = | NA | NA | L-dopa (5.7mg/kg/day); 1, 1. A little improvement, stopped due to LOI | | Haloperidol (0.016mg/kg/day); 8, 1. NA, stopped due to AE | |  |  |
| 27 | 7 | ++ | 12 | 3 | TBZ (12.5mg/day); 12.6, 3. Improvement, continued | | | |  |  |
| 28 | NA | NA | NA | 4 | L-dopa (NA); NA, NA. NA, continued | | TBZ (25mg/12h); 6,2. Without effect, stopped due to LOI | | |  |
| 29 | 2 | = | 2 | 3 |  |  |  |  |  |  |
| 30 | 0.70 | = | 7 | 3 | MFD (15mg/day); 9, 6. NA, continued | | | | |  |
| 31 | 2 | ++ | 5 | NA | Levothyroxine (50ug/day); 5, 10. Improvement, continued | | | | |  |
| 32 | 4 | = | NA | 3 | TBZ (30mg/day); 7,8. Improvement, continued | CZP (NA); 9, NA. Improvement, stopped due to LOI | VPA (NA); 9, NA. Improvement, stopped due to LOI | Trazodone (NA); 9, NA. Improvement, stopped due to LOI |  |  |
| 33 | 3 | ++ | NA | NA | TBZ (NA); NA, NA. Without effect, NA | | | | |  |
| 34 | NA | ++ | NA | 3 | MFD (NA); NA, NA. NA, continued | | | | |  |
| 35 | 1 | ++ | NA | 3 | TBZ (1.5mg/kg/day); 2, 0.3. Without improvement, stopped due to LOI | | L-dopa (7.5mg/kg); 2, 0.1. Worsening, stopped due to AE | | Lisdexanfetamina (NA); NA, NA. NA, continued |  |
| 36 | 0.70 | ++ | 4 | 3 | L-dopa (25mg/8h); 4, 8. Improvement, continued | | | | |  |
| 37 | 2.67 | = | 4 | 3 | CBZ (6.7mg/kg/day); 3, 0.5. Without effect, stopped due to AE and LOI | | L-dopa (12mg/kg/day); 3, 0.5. Without effect, stopped due to AE and LOI | |  |  |
| 38 | 5 | = | NA | 4 | THP (10mg/day); 7,4. Improvement, continued | | MFD (NA); NA, NA. Without effect, continued | |  |  |
| 39 | 0.50 | = | 5 | 3 |  |  |  |  |  |  |
| 40 | 5 | = | 9 | 3 |  |  |  |  |  |  |
| 41 | 0.08 | ++ | 9 | 3 |  |  |  |  |  |  |
| 42 | 1 | ++ | 11 | 3 |  |  |  |  |  |  |
| 43 | 4 | NA | NA | 3 | L-dopa (62.5mg/12h); 4, 6. Improvement, continued | | TBZ (8mg/12h); 5, NA. Without effect, stopped due to LOI | |  |  |
| 44 | 1 | NA | NA | 3 | L-dopa (3.5mg/kg/day); 6, 4. Much improvement, continued | | | | |  |
| 45 | 1 | ++ | 8 | NA |  |  |  |  |  |  |
| 46 | 2 | = | 6 | 3 | TBZ (6.25mg/kg/12h); 6, 0. Without effect, stopped due to AE | | L-dopa (100 mg/12h); 6, 2. Improvement, stopped due to non-specified causes | |  |  |
| 47 | 1 | = | 5 | NA | L-dopa (1mg/kg/day); 4, 1. A little improvement, stopped due LOI and AE | | THP (1mg/kg/day); 5, 2. A little improvement, stopped due to LOI and adhesion problems | | MFD (10-15mg/day); 6, 3. Without effect, continued |  |
| 48 | 5.5 | = | 8 | 3 |  |  |  |  |  |  |
| 49 | 3 | = | NA | 3 | TBZ (2.3mg/kg/day); 6,1. Without effect, stopped due to LOI | | | | |  |
| 50 | 3 | ++ | 6 | 3 |  |  |  |  |  |  |
| 51 | 1 | = | 4 | 4 | L-dopa (3mg/kg/day); 6, 2. Improvement, continued | | | | |  |
| 52 | 0.80 | = | 4 | 3 |  |  |  |  |  |  |
| 53 | 4 | NA | NA | 3 |  |  |  |  |  |  |
| 54 | NA | -- | NA | NA |  |  |  |  |  |  |
| 56 | 2.50 | -- | 4 | 4 |  |  |  |  |  |  |
| 57 | 2 | ++ | 5 | NA |  |  |  |  |  |  |
| 59 | 0.50 | = | 4 | 4 |  |  |  |  |  |  |
| 60 | 2 | = | 4 | 4 |  |  |  |  |  |  |
| 62 | 2 | NA | NA | 3 | TBZ (1mg/kg/day);2, 2. NA, continued | | MFD (20mg/day); 4, 1. Much improvement, continued | | |  |
| 63 | 2.17 | = | 3 | 3 |  |  |  |  |  |  |
| 64 | 1 | = | 12 | 3 |  |  |  |  |  |  |
| 65 | 0.50 | ++ | 3 | NA |  |  |  |  |  |  |
| 66 | 1 | ++ | 3 | 4 |  |  |  |  |  |  |
| 67 | 1.80 | = | NA | 3 | L-dopa (NA); NA, NA. Without effect, stopped due to LOI | | | | |  |
| 68 | 1.00 | = | NA | 4 | L-dopa (NA); 10, 1. Without effect, stopped due to LOI | | | | |  |

Identification (ID), Non available (NA), Improved (++), Stabilized (=), Worsened (--),No (-), Abnormal Involuntary Movement Scale (AIMS), Methylphenidate (MFD), Clonazepam (CZP), Trihexyphenidyl (THP), Tetrabenazine (TBZ), Valproate (VPA), Lack of Improvement (LOI), Adverse Effect (AE)
