# Supplementary material for: International Registry of NKX2‐1‐Related Disorders: Clinical, Genetic, and Imaging Perspectives
Source: Mov Disord. 2026 Jan 19;41(4):889–900. doi: 10.1002/mds.70187 (PMC13067339; doi:10.1002/mds.70187)
Supplement: Supplementary file 5 — Table S3. Genetic and in silico data of NKX2‐1 variants in the study cohort. [file MDS-41-889-s010.docx]

**Supplementary Table 3. Genetic and in silico data of *NKX2-1* variants in the study cohort**

| **ID** | **Genetic Variant, Transcript (NM_001079668.3, unless otherwise specified)** | **Protein Effect** | **Exon Location** | **ACMG Classification** | **PhyloP 100** | **Position** | **Provean** | **MutationT** | **SIFT** | **Variant previously published (PMID)** | **gnomAD Exomes, gnomAD Genomes** | **CADD** | **De novo (inherited from)** |
| --- | --- | --- | --- | --- | --- | --- | --- | --- | --- | --- | --- | --- | --- |
| 1 | *NKX2-1*: c.612C>A (p.Tyr204*), | Nonsense | 3 - HB | P (PVS1, PM2, PP5) | 1,44 | 36517872 | NA | Uncertain | NA | - | 0%, 0% | NA | NA |
| 2 | *NKX2-1*: c.338G>A (p.Trp143Cysfs*23), | Nonsense | 2 | LP (PVS1, PM2) | 5,956 | 36519020 | NA | Uncertain | NA | 30186310* | 0%, 0% | NA | NA |
| 3 | *NKX2-1*: c.338G>A (p.Trp143Cysfs*23), | Nonsense | 2 | LP (PVS1, PM2) | 5,956 | 36519020 | NA | Uncertain | NA | 30186310* | 0%, 0% | NA | - (f) |
| 4 | *NKX2-1*: 347-bp AluYa5 insertion with a 65-bp poly-A tail followed by a 16-bp duplication of the pre-insertion wild-type sequence (ENST00000354822.7:c.556_557insAlu541_556dup) | Alu Retrotransposition | 3 | NA | NA | NA | NA | NA |  | 36420574* | 0%, 0% | 32 | + |
| 5 | NKX2-1: c.463+2T>C/ (p.?) | Splicing – NC | Intron - SR | LP (PVS1, PM2) | 6,782 | 36518983 | NA | Uncertain | NA | - | 0%, 0% | NA | + |
| 6 | *NKX2-1:* c.348delC/ (p.Cys117Alfs*8) | Frameshift | 2 | LP (PVS1, PM2) | 5,517 | 36988304 | NA | NA | NA | 38916623* | 0%, 0% | NA | - (m) |
| 7 | arr[GRCh37] 14q13.3(36,722,498-36,790,795)x1, 68,3 Kb (gene *MBIP*) | Microdeletion | NA | VOUS (1A, 3A, 4L-40, 5B-5E, ) | NA | NA | NA | NA | NA | - | NA | NA | - (m) |
| 8 | *NKX2-1:*c.736delA/ (p.Met246*), | Nonsense | 3 - HB | P (PVS1, PM2, PS2) | 7,835 | 36517748 | NA | NA | NA | - | 0%, 0% | 24,5 | + |
| 9 | *NKX2-1:* c.612C>A/  (p.Tyr204*) | Nonsense | 3 - HB | P (PVS1, PM2, PP5, PS2) | 2,44 | 36517872 | NA | NA | NA | - | 0%, 0% | NA | - (m) |
| 10 | *NKX2-1:* c.634C>T/ (p.Gln212*) | Nonsense | 3 - HB | LP (PVS1, PM2) | 9,766 | 36517850 | NA | Uncertain | NA | - | 0%, 0% | NA | - (f) |
| 11 | *NKX2-1:*c.204C>G/ (p.Tyr68*) | Nonsense | 2 | P (PVS1,PM2, PP5, PS2) | 7,753 | 36519154 | NA | Uncertain | NA | 21982616 | 0%, 0% | 41 | NA |
| 12 | *NKX2-1:* c.342C>G/ (p.Tyr114*), NM_003317.3 | Nonsense | 2 | LP (PVS1, PM2) | 2,675 | 36519016 | NA | Uncertain | NA | - | 0%, 0% | NA | - (NA) |
| 13 | *NKX2-1:* c.612C>A/ (p.Tyr204*) | Nonsense | 3 - HB | P (PVS1, PM2, PP5) | 144% | 36517872 | NA | Uncertain | NA | - | 0%, 0% | NA | - (m) |
| 14 | *NKX2-1:* c.390C>G/ (p.Tyr130*) | Nonsense | 2 | LP (PVS1, PM2) | NA | 36519058 | NA | Uncertain | NA | 34091414* | 0%, NA | NA | - (NA) |
| 15 | *NKX2-1:* c.738_748del/ (p.Met246IlefsTer189) | Frameshift | 3 | LP (PVS1, PM2) | 7,909 | 36517736 | NA | NA | NA | - | 0%, 0% | NA | NA |
| 16 | *NKX2-1:* c.463+1G>A/ (p.?) | Splicing - NC | Intron - SR | P (PVS1, PM2, PP5, PS2) | 7,271 | 36518984 | NA | Uncertain | NA | 28732825, 21555194* | 0%, 0% | NA | + |
| 17 | *NKX2-1:* c.604C>T/ (p.Gln202*) | Nonsense | 3 | P (PVS1, PM2 , PS2) | 7,895 | 36987085 | NA | NA | NA | - | 0%, 0% | NA | + |
| 18 | *NKX2-1:* c.373+1_373+4del/ (p.?) | Splicing - NC | Intron - SR | LP (PM2, PP3, PP1, PP4) | 7,271 | 36988183 | NA | NA | NA | 24930029, 26196025, 22832740* | 0%, 0% | NA | - (f) |
| 19 | *NKX2-1:* c.373+1_373+4del/  (p.?) | Splicing - NC | Intron - SR | LP (PM2, PP3, PP1, PP4) | 7,271 | 36988183 | NA | NA | NA | 24930029, 26196025, 22832740* | 0%, 0% | NA | - (f) |
| 20 | *NKX2-1:* 347-bp AluYa5 insertion with a 65-bp poly-A tail followed by a 16-bp duplication of the pre-insertion wild-type sequence (ENST00000354822.7:c.556_557insAlu541_556dup) | Alu Retrotransposition | 3 | NA | NA | NA | NA | NA |  | 36420574* | 0%, 0% | 32 | - (f) |
| 21 | arr[GRCh37]14q13.2q21.3(35780664-49392228)x1, 13,6Mb (include *NKX2-1* and other genes 2) | Megadeletion | NA | P (2A-2E, 3B, 4L-4O) | NA | NA | NA | NA | NA | NA | NA, NA | NA | + |
| 22 | *NKX2-1:*c.463+5G>C/ (p.?) | Splicing - NC | Intron - SR | LP (PP3, PP5, PM2) | 5,55 | 36518980 | NA | NA | NA | 24714694 | 0%, 0% | NA | + |
| 23 | *NKX2-1*:c.578A>C/ (p.Lys193Thr) | Missense | 3 - HB | P (PP3, PM2, PS2) | 7,835 | 36517906 | P supp. | Uncertain | P supp. | 30009132* | 0%, 0% | 25,2 | + |
| 24 | *NKX2-1:* c.1204dupT/ (p.*402Leuexl*36) | Frameshift | 3 | LP (PM4, PS3, PM2) | 9 | 36.517.279 | NA | NA | NA | - | 0%, 0% | NA | - (f) |
| 25 | *NKX2-1:* c.342C>G/ (p.Tyr114*), NM_003317.3 | Nonsense | 2 | LP (PVS1, PM2) | 2,675 | 36519016 | NA | Uncertain | NA | - | 0%, 0% | NA | - (NA) |
| 26 | arr[GRCh38]14q13.3(35404289_38723530)x1, 3,32 Mb (include *NKX2-1* and other genes 3) | Microdeletion | NA | P (2A-2E, 4L-40) | NA | NA | NA | NA | NA | 29477862* | NA, NA | NA | + |
| 27 | *NKX2-1:*c.622C>T/ (p.Arg208) | Nonsense | 3 | P (PVS1, PM2, PP5) | 1,835 | 36517862 | NA | Uncertain | NA | 17765926 | 0%, 0% | NA | + |
| 28 | *NKX2-1:*c.714G>A/ (p.Trp238*) | Nonsense | 3 - HB | P (PVS1, PP5, PM2) | NA | 36986975 | NA | Uncertain | NA | 36517770 | 0%, 0% | NA | - (f) |
| 29 | c.(?_103)_(*881_?)del, 3.8 Kb (genes *NKX2*-1, *SFTA3*, *NKX2-1-AS1*)/(p.?) | Microdeletion | NA | P (2A-2E, 5D) | NA | NA | NA | NA | NA | - | NA, NA | NA | - (f) |
| 30 | *NKX2-1:*c.638G>A/ (p.Arg243His) | Missense | 3 - HB | P (PM5, PP3, PM1, PM2, PP5, PS2) | 7,726 | 36517756 | P supp. | Uncertain | P supp. | - | 0%, 0% | 31 | + |
| 31 | *NKX2-1:*c.766C>T/ (p.Gln256*) | Nonsense | 3 | LP (PVS1, PM2) | 9,766 | 36986923 | NA | Uncertain | NA | - | 0%, 0% | NA | - (m) |
| 32 | arr[GRCh37] 14q13.1q13.3(35,072,706-36,815,991)x1, 1,7 Mb (include *MBIP* and other genes 4) | Microdeletion | NA | P (2AE-2E, 4L-4º) | NA | NA | NA | NA | NA | - | NA, NA | NA | + |
| 33 | *NKX2-1:*c.632A>G/  ( p.Asn211Ser) | Missense | 3 | LP (PM2, PM1, PP1, PP4) | 6,093 | 36517852 | Uncertain | Uncertain | P supp. | 38757609* | 0%, 0% | 5,46 | - (f) |
| 34 | *NKX2-1:* c.739_746del/ (p.Lys247Glyfs*189) | Frameshift | 3 | LP (PVS1, PM2) | NA | NA | NA | NA | NA | 36054588 | 0%, 0% | NA | - (f) |
| 35 | *NKX2-1:*c.524C>A/ (p.Ser175*) | Nonsense | 3 | P (PVS1, PM2, PP5, PS2) | NA | 36517960 | NA | Uncertain | NA | 18788921, 27066577* | 0%, 0% | NA | + |
| 36 | *NKX2-1*:c.612C>A/  (p. Tyr204*), | Nonsense | 3 | P (PVS1, PM2, PP5, PS2) | 2,44 | 36517872 | NA | NA | NA | - | 0%, 0% | NA | + |
| 37 | *NKX2-1:*c.727C>A/ (p.Arg243Ser) | Missense | 3 - HB | P (PS1, PM5, PP3, PM1, PM2, PP5, PS2) | 9,766 | 36517757 | P supp. | Uncertain | P supp. | 15955952, 24453141, 26723978, 11971878 | 0%, 0% | 34 | + |
| 38 | *NKX2-1:* c.272del/  (p. p.His91Profs*10) | Frameshift | 2 | LP (PVS1, PM2) | 4,803 | 36519176 | NA | NA | NA | - | 0%, 0% | NA | + |
| 39 | *NKX2-1*:c.463+2T>C/(p.?), | Splicing - NC | Intron - SR | LP (PVS1, PM2) | 6,782 | 36518983 | NA | Uncertain | NA | - | 0%, 0% | NA | NA |
| 40 | *NKX2-1:* c.291292insCCACGGCGCCGTCACCGCCGCC/(p.?) | Frameshift | 2 | NA | NA | NA | NA | NA | NA | - | NA, NA | NA | NA |
| 41 | *NKX2-1:* c.211del/  (p.Tyr71ThrfsTer30) | Frameshift | 2 | P (PVS1, PM2, PS2) | 6,999 | 36519237 | NA | NA | NA | - | 0%, 0% | NA | + |
| 42 | arr[GRCh38] 14q13.3(36875728_37157010)x1, 281.3 kb (genes PAX9, *SLC25A21* and *MIR4503*) | Microdeletion | NA | P (1A, 2A, 2B, 2D, 3A, 4L) | NA | NA | NA | NA | NA | - | NA, NA | NA | + |
| 43 | *NKX2-1:* c.714G>A/ (p.Trp238*) | Nonsense | 3 - HB | P (PVS1, PP5, PM2) | NA | 36986975 | NA | Uncertain | NA | 36517770 | 0%, 0% | NA | - (m) |
| 44 | *NKX2-1:* c.632A>G/ (p.Lys211Arg) | Missense | 3 - HB | LP (PM1, PP3, PM2, PS2) | 6,093 | 36517852 | Uncertain | Uncertain | P supp. | 38757609 | 0%, 0% | 32 | + |
| 45 | *NKX2-1:*c.593T>G/ (p.Phe198Cys) | Missense | 3 - HB | P (PM2, PP3, PM1, PS2) | 7,29 | 36987096 | P moderate | Uncertain | P supp. | - | 0%, 0% | 33 | - (m) |
| 46 | *NKX2-1:* c.733A>T/  (p.Lys245*) | Nonsense | 3 - HB | P (PVS1, PM2, PP5) | 7,835 | 36517751 | NA | Uncertain | NA | - | 0%, 0% | 40 | -(f) |
| 47 | *NKX2-1:* c.429_434delinsTA/ (p.Trp143Cysfs*22) | Frameshift | 2 | LP (PVS1, PM2) | 7,305 | 36519014 | NA | NA | NA | - | 0%, 0% | NA | -(m) |
| 48 | *NKX2-1:*c.206_207delCG/ (p.Ala69Glyfs*369) | Frameshift | 2 | LP (PVS1, PM2) | 7,511 | 36519240 | NA | NA | NA | - | 0%, 0% | NA | NA |
| 49 | *NKX2-1:*c.464-1G>A/(p.?) | Splicing - NC | Intron - SR | LP (PVS1, PM2) | 6,022 | 36987226 | NA | Uncertain | NA | 22155464 | 0%, 0% | NA | NA |
| 50 | *NKX2-1:*c.606G>C/ (p.Gln202His) | Missense | 3 - HB | P (PM5, PP3, PM1, PM2, PP5, PS2) | 3,282 | 36517878 | P supp. | Uncertain | P supp. | 26723978, 15955952, 24453141, 11971878 | 0%, 0% | 25,1 | + |
| 51 | *NKX2-1:* c.642C>A/ (p.Tyr244*) | Nonsense | 3 - HB | P (PVS1, PM2, PS2) | 6,648 | 36986957 | NA | NA | NA | 24930029 | 0%, 0% | NA | + |
| 52 | *NKX2-1:* c.489del/ (p.Ser163Argfs*3) | Frameshift | 3 | P (PVS1, PM2, PS3) | 0,642 | 36517995 | NA | Uncertain | NA | 19336474, 15289765 | 0%, 0% | NA | -(f) |
| 53 | *NKX2-1:*c.598C>T/ (p.Gln200*) | Nonsense | 3 - HB | LP (PVS1, PM2) | 9,766 | 36517886 | NA | Uncertain | NA | - | 0%, 0% | NA | -(NA) |
| 54 | *NKX2-1:* c.727C>A/ (p.Arg243Ser) | Missense | 3 - HB | P (PS1, PM5, PP3, PM1, PM2, PP5, PS2) | 9,766 | 36517757 | P supp. | Uncertain | P supp. | 15955952, 24453141, 26723978, 11971878 | 0%, 0% | 34 | NA |
| 55 | *NKX2-1:*c.972_973del/ (p.Gln325GlyfsTer113) | Frameshift | 3 | P (PVS1, PM2, PS2) | 3,596 | 36517511 | NA | NA | NA | - | 0%, 0% | NA | + |
| 56 | arr[GRCh37] 14q13.1q21.1 (35085096_37969701)x1 2,88 Mb (include *NKX2-1* and other genes 5) | Microdeletion | NA | P (1A, 2A-2E, 4L-4º) | NA | NA | NA | NA | NA | - | NA, NA | NA | + |
| 57 | *NKX2-1:*c.718C>T/ (p.Gln240Ter), NA | Nonsense | 3 | LP (PVS1, PM2) | 7,908 | 36517766 | NA | NA | NA | - | 0%, 0% | NA | NA |
| 58 | arr[GRCh37] 14q12-q21.1 (27654804-42422184)x1, 14.7 Mb (include *NKX2-1* and other genes 6) | Megadeletion | NA | P (1A, 2A-2E, 3C, 4L-4) | NA | NA | NA | NA | NA | - | NA, NA | NA | NA |
| 59 | *NKX2-1:* c.489del/ (p.Ser163Argfs*3) | Frameshift | 3 | P (PVS1, PM2, PS3) | 0,642 | 36517995 | NA | Uncertain | NA | 19336474, 15289765 | 0%, 0% | NA | + |
| 60 | *NKX2-1:* c.567_582del (p.Pro190Glyfs*33), NA | Frameshift | 3 - HB | LP (PVS1, PM2) | 9,646 | 36517902 | NA | NA | NA | - | 0%, 0% | NA | + |
| 61 | *NKX2-1:*c.562_583del/ (p.Ser188Glyfs*33) | Frameshift | 3 | P (PVS1, PM2, PS2) | 9,646 | 36517901 | NA | NA | NA | - | 0%, 0% | NA | + |
| 62 | arr[GRCh37] 7q11.21, add(13)(q14), add(14)(q11.2), add(22)(q13.1) | Translocation | NA | NA | NA | NA | NA | NA | NA | - | NA, NA | NA | NA |
| 63 | *NKX2-1:*c.463+5G>C/(p.?) | Splicing - NC | Intron - SR | P (PP3, PP5, PM2, PS2) | 5,501 | 36518980 | NA | NA | NA | 24714694 | 0%, 0% | NA | NA |
| 64 | *NKX2-1:*c.733A>T/  (p. Lys245*) | Nonsense | 3 - HB | P (PVS1, PM2, PP5) | 7,835 | 36517751 | NA | Uncertain | NA | - | 0%, 0% | NA | - (f) |
| 65 | *NKX2-1:*c.904del/ (p.Ala302Argfs*79) | Frameshift | 3 | LP(PVS1, PM2) | 2,985 | 36986784 | NA | NA | NA | - | 0%, 0% | NA | - (m) |
| 66 | *NKX2-1*:c.646A>T/ (p.Met246Leu) | Missense | 3 | LP(PM2, PP3, PM1, PS2) | 7,809 | 36517748 | Benign Moderate | Uncertain | Benign Moderate | - | 0%, 0% | 4,178 | + |
| 67 | *NKX2-1*:c.464-2A>G/(p.?) | Splicing - NC | Intron - SR | P(PVS1, PM2, PS3) | 6,128 | 36518022 | NA | Uncertain | NA | 19336474, 15289765 | 0%, 0% | NA | NA |
| 68 | *NKX2-1*:c.1073dup/ (p.Asp359GlyfsTer80) | Frameshift | 3 | P(PVS1, PM2, PS2) | 5,741 | 36517411 | NA | NA | NA | - | 0%, 0% | NA | + |

Identification (ID), American College of Medical Genetics and Genomics (ACMG), MutationTaster (MutationT), Sorting Intolerant From Tolerant (SIFT), PubMed Identifier (PMID), gnomAD (Genome Aggregation Database), Combined Annotation Dependent Depletion (CADD), Non available (NA), Homeobox (HB), Yes (+), No (-), NC (Non coding), SR (Splicing region), Pathogenic (P), Probable Pathogenic (PP), Variant of Unknown Significance (VOUS), Pathogenic Very Strong 1 (PVS1), Pathogenic Moderate 2 (PM2), Pathogenic Supporting 5 (PP5), Pathogenic Strong 2 (PS2), Pathogenic Supporting 3 (PP3), Pathogenic Strong 1 (PS1), Pathogenic Moderate 5 (PM5), Pathogenic Moderate 1 (PM1), Pathogenic Supporting 1(PP1, Pathogenic Supporting 4 (PP4), Father (f), Mother (m), supporting (supp.),

^*^ (same individual previously reported)

^1^ The proband's WES was analyzed for MEIs. A candidate MEI in NKX2-1 underwent optimized SS after plasmid cloning. Functional studies exploring NKX2-1 haploinsufficiency at RNA and protein levels were performed.

^2^ Other genes included: *SNORA101B, LINC00609, LOC101927418, LINC02302, BRMS1L, MIA2, FSCB, FBXO33, PRPF39, MIPOL1, FOXA1, LOC105370457, LINC00648, PSMA6, LINC00517, SSTR1, GEMIN2, LINC02303, RALGAPA1, SEC23A, TTC6, MBIP, SLC25A21, INSM2, SNORD127, FANCM, FKBP3, PTCSC3, LINC02315, MIR4503, SFTA3, PNN, NKX2-1-AS1, LINC00639, MDGA2, C14orf28, RPL10L, MIR548Y, LOC112268136, LOC100288846, MIS18BP1, CLEC14A, KLHL28, LINC00871, NKX2-1, NFKBIA, NKX2-8, LRFN5, SEC23A-AS1, TOGARAM1, PAX9, SLC25A21-AS1, LOC105370473, TRAPPC6.*

^3^ Other genes included: *PAX9, SLC25A21, NFKBIA, SRP54, RALGAPA1, PSMA6, FOXA1 (HNF3), MIPOL1, BRMS1L, MBIP, SSTR1, PPP2R3C (G5PR), MRPP3 (KIAA0391), NKX2-8, INSM2 (IA6), SFTA3, CLEC14A, C14ORF19 and FAM177A1 (C14ORF24).*

^4^ Other genes included: *RALGAPA1, NFKBIA, FAM177A1, PRORP, CFL2, SNX6, PSMA6, PPP2R3C, PTCSC3, SNORA101B, BAZ1A, IGFP1P1, SRP54, INSM2, LOC101927178, SRP54-AS1, LOC112268124, LINC00609, BRMS1L,* 14q13.2.

^5^ Other genes included: *PAX9, NFKBIA, RALGAPA1, PRORP, FAM177A1, SLC25A21, CFL2, INSM2, NKX2-8, PSMA6, MIPOL1, SRP54, PTCSC3, PPP2R3C, SLC25A21-AS1, BAZ1A, SNX6, MBIP, SNORA101B, MIR4503, LOC101927178, IGBP1P1, BRMS1L, LINC00609, SRP54-AS1, NKX2-1-AS1, SFTA3, LOC112268124.*

^6^ Other genes included: *PAX9, FOXG1, RALGAPA1, NFKBIA, NUBPL, TRAPPC6B, COCH, PRKD1, ARHGAP5, FAM177A1, SLC25A21, SPTSSA, PRORP, SEC23A, AP4S1, CFL2, LOC100288846, AKAP6, LINC02313, FBXO33, LINC01551, PNN, NKX2-1-AS1, NPAS3, PSMA6, MIA2, LINC02315, PPP2R3C, LINC02282, SSTR1, PTCSC3, HEATR5A, SLC25A21-AS1, LOC105370457, EGLN3, FOXG1-AS1, SNX6, SNORA101B, DTD2, ARHGAP5-AS1, MIR624, BAZ1A, LOC101927124, HECTD1, MIR4503, SCFD1, GEMIN2, LINC00517, LOC728755, IGBP1P1, SRP54, INSM2, SEC23A-AS1, GPR33, LOC101927178, TTC6, RNU6-8, LINC00639, LINC00645, FOXA1, SRP54-AS1, MIR3171, LOC112268124, LRFN5, LOC100506071, EAPP, LINC00609, MIPOL1, G2E3-AS1, SNORA89, BRMS1L, RNU6-7, STRN3, MBIP, NKX2-8, CLEC14A, LOC112268136, SFTA3, G2E3,* 14q13.2.
