# Supplementary material for: International Registry of NKX2‐1‐Related Disorders: Clinical, Genetic, and Imaging Perspectives
Source: Mov Disord. 2026 Jan 19;41(4):889–900. doi: 10.1002/mds.70187 (PMC13067339; doi:10.1002/mds.70187)
Supplement: Supplementary file 6 — Table S4. Frequency and percentage of NKX2‐1‐related disorders individuals followed up by each medical specialist. [file MDS-41-889-s003.docx]

| **Follow up frequency** | **Neurologists** | **Pulmonologists** | **Endocrinologist** |
| --- | --- | --- | --- |
| Quarterly | 4/61 (6.56%) | 3/57 (5.26%) | 3/57 (5.26%) |
| Biannually | 24/61 (39.34%) | 13/57 (22.81%) | 16/57 (28.07%) |
| Annually | 23/61 (37.7%) | 6/57 (10.53%) | 27/57 (47.37%) |
| Once every year and a half | 3/61 (4.92%) | 1/57 (1.75%) | - |
| Biennially | 2/61 (3.28%) | 1/57 (1.75%) | - |
| Every five years | - | 2/57 (3.51%) | 1/57 (1.75%) |
| No follow up | 5/61 (8.20%) | 31/57 (54.39%) | 10/57 (17.55%) |

**Supplementary Table 4. Frequency and percentage of *NKX2-1*-related disorders individuals followed up by each medical specialist**
