# Supplementary figures and images for: International Registry of NKX2‐1‐Related Disorders: Clinical, Genetic, and Imaging Perspectives
Source: Mov Disord. 2026 Jan 19;41(4):889–900. doi: 10.1002/mds.70187 (PMC13067339; doi:10.1002/mds.70187)

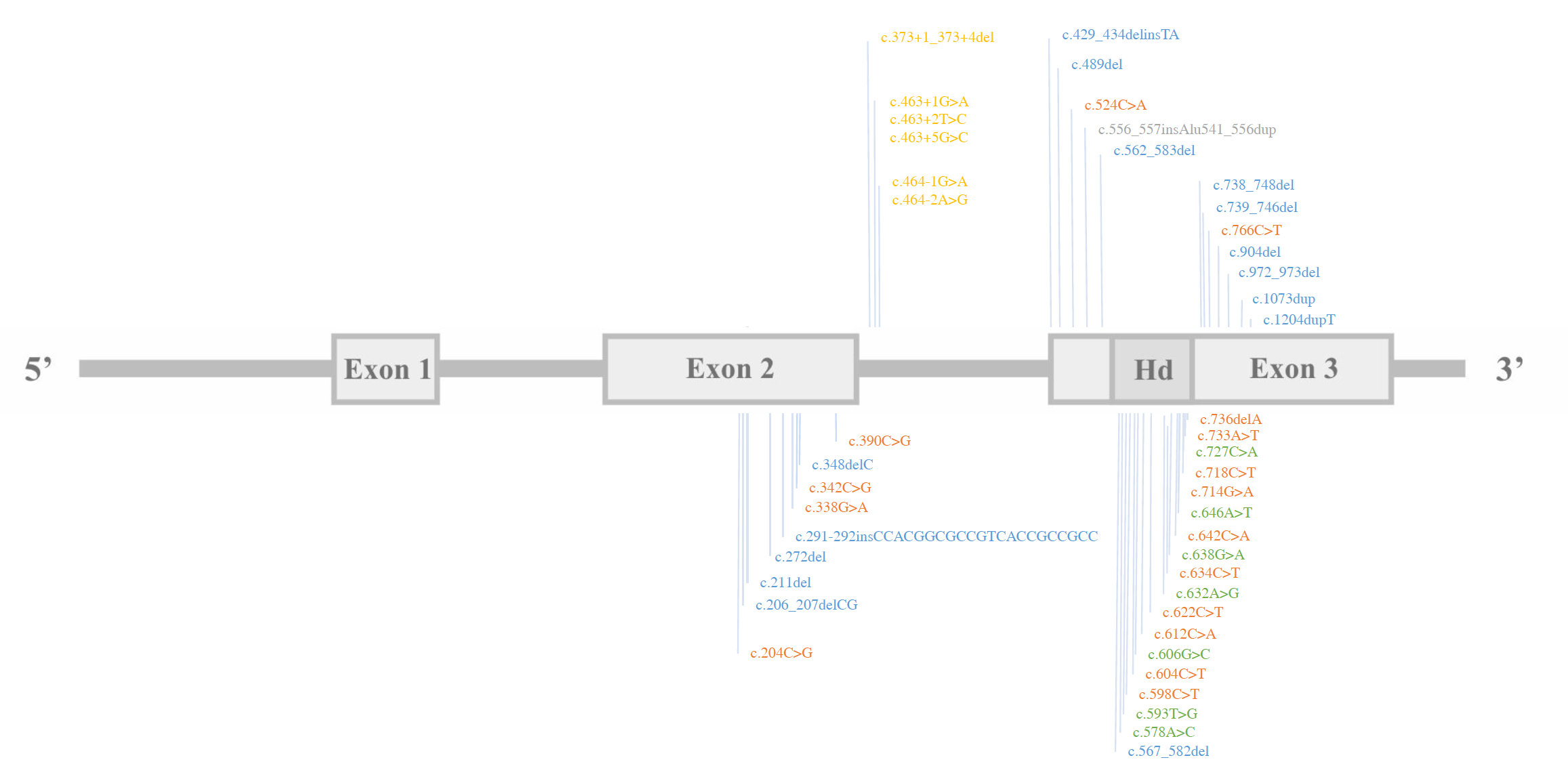

Supplement: Supplementary file 7 — Figure S1. Distribution of 45 NKX2‐1‐single nucleotide variants relative to the functional domains of NKX2‐1 isoform 2 (RefSeq NM_003317.3). Distal and proximal promoters are indicated by arrows. Functional domains are shown in dark grey: TN (tinman domain), HD (homeodomain), and NK2 (NK2‐specific domain). Nonsense variants (orange), frameshift variants (blue); missense variants (green); splicing – non‐coding variants (yellow), and Alu retrotransposition events (grey). [file MDS-41-889-s009.png]
